# Vitamin auxotrophies shape microbial community assembly on model marine particles

**DOI:** 10.1093/ismejo/wraf184

**Published:** 2025-08-20

**Authors:** Rachel Gregor, Gabriel T Vercelli, Rachel E Szabo, Matti Gralka, Ryan C Reynolds, Evan B Qu, Naomi M Levine, Otto X Cordero

**Affiliations:** Department of Civil and Environmental Engineering, Massachusetts Institute of Technology, Cambridge, MA, 02139-4307, United States; Department of Civil and Environmental Engineering, Massachusetts Institute of Technology, Cambridge, MA, 02139-4307, United States; Department of Civil and Environmental Engineering, Massachusetts Institute of Technology, Cambridge, MA, 02139-4307, United States; Systems Biology Group, Amsterdam Institute for Life and Environment (A-LIFE) and Amsterdam Institute of Molecular and Life Sciences (AIMMS), Vrije Universiteit Amsterdam, 1081 HZ, Amsterdam, The Netherlands; Department of Biological Sciences, University of Southern California, Los Angeles, CA, 90089-0371, United States; Department of Civil and Environmental Engineering, Massachusetts Institute of Technology, Cambridge, MA, 02139-4307, United States; Department of Biological Sciences, University of Southern California, Los Angeles, CA, 90089-0371, United States; Department of Civil and Environmental Engineering, Massachusetts Institute of Technology, Cambridge, MA, 02139-4307, United States

**Keywords:** B vitamins, auxotrophies, community assembly, marine snow, cross-feeding, chitin

## Abstract

Microbial community assembly is governed by the flow of carbon sources and other primary metabolites between species. However, central metabolism represents only a small fraction of the biosynthetic repertoire of microbes: metabolites such as antimicrobial compounds, signaling molecules, and co-factors are underexplored in their potential to shape microbial communities. Here, we focus on B vitamin exchange in marine bacterial communities that degrade polysaccharides, a key component of particulate organic matter. We found that in a screen of 150 natural isolates, almost a third were auxotrophs for one or more B vitamins. By measuring physiological parameters such as uptake affinities and comparing those to ambient seawater concentrations, we showed that marine bacteria live at the edge of vitamin limitation in the environment. To understand how auxotrophs survive in the open oceans, we used our experimental data to model vitamin cross-feeding on particles through both secretion and lysis. Our results highlight the importance of vitamin auxotrophies in shaping microbial community assembly and succession, adding another layer of complexity to the trophic structure of particle-associated communities.

## Introduction

In the vast ocean, particles of organic matter serve as hubs of microbial activity and interactions [1, 2]. These particles, termed marine snow, are colonized and degraded by specialized bacteria that release sugars and other substrates such as organic acids, supporting the growth of cross-feeding bacteria [3, 4]. Particle-associated communities harbor a dense network of interactions, through quorum sensing [5], antibiotics [6], and increased potential for horizontal gene transfer [7]. Metabolic dependencies frequently evolve in these communities, for example through the loss of genes to produce siderophores [8] or hydrolytic enzymes [9]. However, this is a high-risk, high-reward strategy: auxotrophic bacteria that lose the ability to produce essential nutrients gain a fitness advantage, but only as long as the nutrients remain available in the environment [10]. In bacterial communities, these metabolic dependencies are hidden forces that shape community assembly and the balance between cooperation and competition in natural environments.

Vitamins are uniquely suited as a case study for metabolic dependencies in marine microbial community assembly. These metabolites are highly conserved and required only in trace amounts, as coenzymes for central metabolic reactions. Vitamin auxotrophies are predicted to be prevalent among marine microbes [11–16], resulting in dependencies on exogenous sources of vitamers (vitamins and related compounds, including biosynthetic precursors and degradation products). Vitamin cross-feeding plays a key role in algal–bacterial interactions [17] and across environments [18–20], but has not been studied in marine particle-associated communities. It remains unclear whether the trace levels of vitamins in seawater are limiting or sufficient for auxotroph growth [15, 21–23]. If vitamins are indeed limiting, then biotic vitamin exchange is likely to be an important driver in community dynamics. This is especially relevant in the resource-rich particle environment, where other nutrients may not be limiting, and fast growth is favored.

Here, we examine the role of B vitamins in the assembly of marine particle-associated bacterial communities, focusing on chitin-degrading communities. We combine natural seawater experiments with a large-scale characterization of 150 isolates from marine polysaccharides, to provide insight into vitamin exchange in particle communities through the phenotypes of naturally co-existing, environmentally relevant, and taxonomically diverse bacteria. We find that auxotrophies are prevalent and that B vitamins are likely limiting in the particle environment, demonstrating the importance of auxotrophies in community assembly and function.

## Materials and methods

### Seawater microcosms

Nearshore coastal seawater was collected from Nahant, MA, on 21 October 2021, and filtered sequentially with 63 μm and 5 μm filters. Hydrogel chitin particles (New England Biolabs, #E8036L) were washed in sterile artificial seawater (Sigma-Aldrich, #S9883) and filtered through 100 μm mesh filters. The flowthrough was then passed through a 40 μm filter, and the particles captured on the filter were resuspended in artificial seawater. The particles were added to 50 ml aliquots of filtered seawater, for a final concentration of ~750 particles per tube (15 particles/ml). To half of the tubes, vitamins were added to the same final concentrations present in marine biological laboratory (MBL) minimal growth medium (Table 1; see below for details on medium components). The tubes were rotated end over end at room temperature over five days. Every 12 hours, we harvested three replicate tubes from each condition and separated the chitin particles and associated bacterial community using a neodymium magnet. The seawater supernatant was removed with a serological pipette, leaving ~1 ml seawater and beads. The seawater+beads fraction was transferred to an Eppendorf tube and 0.5 ml of artificial sea salt solution was added. The samples were frozen at −20°C.

**Table 1:** Vitamin concentrations in standard MBL with added vitamins (MBL + V), and after 3× 1:40 dilutions (i.e. 1:64 000 dilution) in MBL with no added vitamins (MBL–V). *Note*: The dilution calculations are based on the initial concentrations in MBL + V, and do not account for consumption, production, or loss through discarding cell biomass during dilutions. For auxotrophs, the concentration after three growth-dilution cycles is likely lower, as vitamins would be consumed, but not produced.

**Vitamin**	**Abbreviation**	**MBL + V conc. (M)**	**Final conc. after dilution in MBL–V (M)**
Lipoic acid	ALA	4.85 × 10^−7^	7.57 × 10^−12^
Thiamine hydrochloride	B1	2.97 × 10^−7^	4.63 × 10^−12^
Thiamine pyrophosphate	B1-PP	2.17 × 10^−7^	3.39 × 10^−12^
Riboflavin	B2	2.66 × 10^−7^	4.15 × 10^−12^
Nicotinic acid	B3	8.12 × 10^−7^	1.27 × 10^−11^
Nicotinamide adenine dinucleotide	B3-NAD	1.51 × 10^−7^	2.36 × 10^−12^
Calcium d-pantothenate	B5	4.20 × 10^−7^	6.56 × 10^−12^
Pyridoxine hydrochloride	B6	4.86 × 10^−7^	7.60 × 10^−12^
D-Biotin	B7	1.23 × 10^−7^	1.92 × 10^−12^
Folic acid	B9	2.27 × 10^−7^	3.54 × 10^−12^
4-aminobenzoic acid	B9-PABA	7.29 × 10^−7^	1.14 × 10^−11^
L-ascorbic acid	C	5.68 × 10^−7^	8.87 × 10^−12^
Cyanocobalamin	B12	7.38 × 10^−9^	1.15 × 10^−13^

### DNA extractions and sequencing

Genomic DNA was extracted from two replicate tubes per condition using the Qiagen DNeasy Blood & Tissue Kit. The DNA yield was quantified using the Invitrogen Quant-iT PicoGreen dsDNA Assay, with yields ranging between 0.01 and 0.04 ng/μl. The samples were processed and sequenced by the Microbial Genome Sequencing Center (now SeqCenter) using the Illumina DNA Prep kit and protocol, modified for low concentrations. Paired end sequencing was run on a NextSeq2000 (Illumina), yielding 2 × 151 bp reads. Raw sequencing reads were quality trimmed with Trimmomatic v0.36 [24]. The metagenomes were annotated for taxonomy with phyloFlash v3.3b3 [25] using SILVA 138 [26, 27]. Chitinases were annotated using a custom database of profile hidden Markov models of proteins involved in growth on chitin [28].

### Isolate collection

For a full list of strains, including their taxonomy and isolation details, see Supplementary Dataset S1. Apart from the *Vibrionales* strains YB2 [29] and 1A06, 12B01, and 13B01 [30], all strains were originally isolated from coastal seawater from Nahant, MA, USA, in enrichments containing polysaccharides, including chitin, chitosan, carageenan, agarose, and alginate [3, 4]. All isolates were first enriched in seawater microcosms containing polysaccharides, and therefore selected by the conditions there, before being transferred to rich isolation medium containing vitamins. All strains were originally double-streaked at the time of isolation. From the overall collection, 187 strains were selected, double-streaked again, and arrayed in two 96-well plates and stored in 5% dimethylsulfoxide at −80°C [31].

Because vitamins were included in the rich isolation medium, it is possible that these conditions enriched for vitamin auxotrophs. However, the proportion of auxotrophs found in this study (~30%) is generally in agreement with previous estimates based on genome annotations [11, 12, 14–16]. The selected strains are biased toward robust and relatively fast growers, to facilitate high-throughput screening.

### Media and growth conditions

All experiments with isolates were performed at room temperature or 25°C incubators in liquid culture using MBL minimal medium (see below). For co-cultures and kinetic experiments, frozen individual environmental isolates were streaked onto marine broth (Marine Broth 2216, Difco) agar plates, and single colonies were inoculated into marine broth liquid medium.

MBL medium is a defined marine minimal medium adapted from a protocol from the Marine Biological Laboratory Microbial Diversity course, as follows: 4× sea salt mix (1 l water containing 80 g NaCl, 12 g MgCl_2_·6H_2_O, 0.60 g CaCl_2_·2H_2_O, 2.0 g KCl); 1000× trace mineral mix (1 l 20 mM HCl containing: 2100 mg FeSO_4_·7H_2_O, 30 mg H_3_BO_3_, 100 mg MnCl_2_·4H_2_O, 190 mg CoCl_2_·6H_2_O, 24 mg NiCl_2_·6H_2_O, 2 mg CuCl_2_·2H_2_O, 144 mg ZnSO_4_·7H_2_O, 36 mg Na_2_MoO_4_·2H_2_O; 25 mg NaVO_3_, 25 mg NaWO_4_·2H_2_O, 6 mg Na_2_SeO_3_·5H_2_O; note: NaVO_3_ and NaWO_4_·2H_2_O solids were handled in the chemical hood); 100× nitrogen source (1 M NH_4_Cl); 500× phosphorus source (0.5 M Na_2_HPO_4_); 1000× sulfur source (1 M Na_2_SO_4_); 20× HEPES buffer (1 M HEPES sodium salt, pH 8.2), and a carbon source and vitamins as detailed below. All components were filter sterilized with 0.2 μm filters. Sea salt mix was stored at room temperature; sulfur, nitrogen, carbon, and phosphorus sources were stored in 4°C; stocks of vitamins, trace metals, and HEPES were aliquoted into single use aliquots and frozen at −20°C.

For standard MBL (here labeled MBL + V), a vitamin mix is added to final concentrations as listed in Table 1. MBL–V contains all medium components except for vitamins. For the phenotype characterization, four carbon sources (glucose, glutamine, glycerol, and pyruvate) were added to the MBL medium to support the growth of isolates with different nutrient preferences. Each carbon source was normalized by its number of carbon atoms to achieve a final concentration of 15 mm carbon each (2.5 mm glucose; 3 mm glutamine; 5 mm sodium pyruvate; 5 mm glycerol). For the kinetic growth curves as well as the supernatant/lysate experiments, a single preferred carbon source at 30 mm C was included (glucose for prototroph 1A01, pyruvate for all auxotrophs characterized), to provide carbon in excess and avoid diauxic shifts.

### Vitamin auxotrophy characterization

For the phenotype characterization, the arrayed isolate collection was defrosted and inoculated 1:10 into marine broth liquid medium in 2 ml 96-well plates. After 72 hours, the plates were transferred 1:100 into MBL + V medium for acclimation in minimal medium with vitamins present. After 24 hours, the cultures were transferred 1:40 into standard 96-well plates in two conditions, each in triplicate: MBL + V and MBL–V. Every 24 hours, the cultures were diluted 1:40 and transferred again to the same medium conditions (MBL + V or MBL–V). By the third transfer in the MBL–V condition, the vitamins present from the initial acclimation step had been diluted 1:64 000, to a final concentration of added vitamins ranging from 13 to 0.1 pM (Table 1).

Every 24 hours, bacterial growth was estimated by measuring the optical density at 600 nm (OD_600_) using a TecanSpark plate reader with a stacker module. Then the plates were transferred 1:40 into fresh medium (MBL + V or MBL–V). Plates were incubated at room temperature without shaking. Isolates that did not grow consistently upon being transferred to MBL + V were dropped from the screen, leaving 150 isolates in total.

The isolates were classified as vitamin auxotrophs using the growth data from the third transfer (72 hours). The median OD_600_ value was calculated for both conditions and the growth deficit calculated as follows: (MBL–V – MBL + V)/(MBL–V + MBL + V). A growth deficit value of −.4 was chosen as the cutoff for putative auxotrophs based on the distribution of values, with 52 isolates passing this threshold (Supplementary Fig. S1). The putative auxotrophs were then grown individually and assayed for growth with each vitamin separately, with supplementation of single vitamins as well as mixes of all vitamins but one. Each putative auxotroph was tested at least twice and the results compared. Forty-seven of the putative auxotrophs were confirmed, with the remaining five removed as their phenotype was not consistent.

### Genome analysis

We established the genetic basis of vitamin auxotrophies by comparing the results of the phenotype characterization to the isolate genomes. The sequenced genomes [3, 4, 31] were annotated using eggnog v4.5 (mmseqs mode with default parameters) [32] and dbCAN v2 (diamond mode) [33] for CAZymes. Taxonomy assignment and the creation of phylogenetic trees were performed using the standard workflow in GTDB-tk (version GTDB R202) [34], followed by renaming of taxa falling into the NCBI clades *Vibrionales* and *Alteromonadales* (both assigned *Enterobacterales* by GTDB-tk) to use the more familiar names for those clades [35]. The eggnog annotations were searched for vitamin biosynthesis genes based on previous studies (Supplementary Tables S1 and S2) [15, 16, 36–39]. The same process was followed to annotate reference genomes from proGenomes.

### Growth curves

To determine growth requirements for vitamins, the growth of auxotrophs was measured across vitamin concentrations spanning seven orders of magnitude. A selection of auxotrophs for different vitamins was chosen and grown in the same transfer protocol described above in the phenotype characterization, with two key changes: (i) Each auxotroph was initially grown in marine broth and then MBL + V as specified above, then transferred to eight conditions in total: MBL–V, and seven 10-fold dilutions of a single vitamin in MBL–V. The vitamins were added in each transfer. (ii) After the third transfer, the OD_600_ was measured continuously in the plate reader for 24–36 hours. The resulting growth curves were visualized and the growth rate calculated for each vitamin concentration from the exponential region after log transformation of the OD_600_ values, using the lm function in R. Half-saturation constants based on the growth rates were calculated using the drc package (v3.0-1) in R.

### Co-culture and supernatant experiments

We grew a selection of eight auxotrophs in co-culture, including two dual auxotrophs. The same general growth protocol was used as described above in the phenotype characterization, with the following modifications. Each auxotroph was initially grown in monoculture to high density in marine broth and then MBL + V. The monocultures were all normalized to OD_600_ 0.2, then combined in pairs (encompassing all possible combinations) and diluted 1:40 into MBL–V medium. The co-cultures were grown as described above, with daily 1:40 transfers and OD_600_ measurements. As a control, the same co-cultures were grown in MBL + V medium.

For the supernatant and lysis experiments, exponentially growing cultures of 1A01 were grown in replicate 4 ml volumes in tubes. At the collection timepoints, samples were collected for both supernatants and lysates. For the supernatants, cells were pelleted by centrifugation (6000 g for 10 minutes at room temperature), and the supernatant carefully removed and sterilized by 0.2 μm filtration to remove any remaining cells. The supernatants were immediately frozen at −80°C. For the lysates, the cultures (containing cells suspended in supernatant) were immediately frozen at −80°C. On the day of the experiment, all lysate samples were defrosted and lysed with a microtip probe sonicator on ice (Misonix Sonicator 3000, 2 minutes total of sonication at power setting 5, in intervals of 30 seconds on/60 seconds off). The supernatants were also defrosted, and both supernatants and lysates were stored at 4°C throughout the experiment. The experiments were performed using the standard protocol described above. The supernatants and lysates were mixed in a 1:1 ratio with fresh MBL–V medium, with MBL + V medium serving as a control. The cultures were grown as described above, with daily 1:40 transfers and OD_600_ measurements for 3 days.

### Modeling, statistical analysis, and data visualization

The degrader and substrate parameters were based on previous work on *Vibrio.* 1A01 [40–42], the isolate used in supernatant and lysate experiments. Vitamin half-saturation constants were measured from this work, and approximate vitamin yields (cells per vitamin molecules) were estimated from the literature [16, 43, 44]. The half-saturation constant for acetate was estimated based on published measurements in *Escherichia coli* [45–47]. A detailed description of the model of vitamin-limited growth on particles is available in the SI text. Numerical simulations of the model were run using the py-pde package [48] v0.32.1 in Python v5.3.3. Statistical analyses were in general done using R v4.0.2 [49]. Plots were created using the ggplot2 package v3.3.3 [50] and ggtree v2.4.1 [51]. Genomes were visualized using clinker [52]. Statistical analysis of the proGenomes database was performed in Mathematica 13.2, with all *P*-values derived from unconstrained linear model fits.

## Results

### Vitamins are a limiting nutrient in coastal seawater microcosms

To determine if vitamins are limiting in particle-associated communities, we examined the effect of vitamin supplementation on coastal seawater microcosms incubated with model particles. As a simplified model for natural organic matter particles, we used particulate chitin, an abundant marine polysaccharide degradable only by specialized bacteria. We have previously shown that chitin particles are colonized by marine bacteria from the surrounding seawater in successive waves of degraders and cross-feeders [3, 4]. To test whether vitamins play a role in structuring these complex microbial communities, we incubated coastal seawater from Nahant, MA with chitin particles and supplemented half of the samples with a mixture of vitamins formulated for marine culture medium (Table 1; see Methods for details). Twice daily, we evaluated the impact of vitamins on community assembly by harvesting the particle-associated bacterial community and sequencing the samples using shotgun metagenomics.

**Figure 1:**
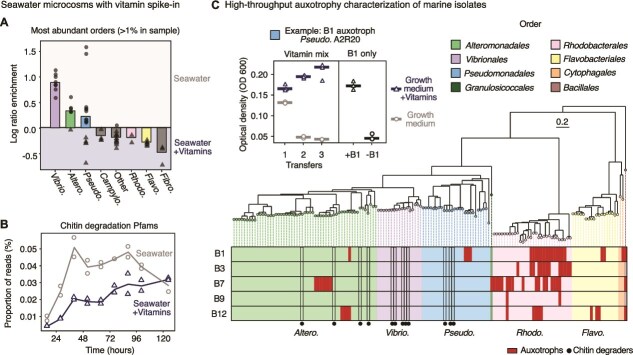
Coastal seawater communities are vitamin-limited and vitamin auxotrophies are prevalent. (A) Coastal seawater collected at Nahant, MA, was incubated with chitin particles, with and without a vitamin mix spike-in. The addition of vitamins led to differences in taxonomy at the order level. Each data point represents a taxon that reached at least 1% abundance in one or more time points. (B) Chitin degradation proteins families (Pfams) were depleted in the vitamin-supplemented seawater condition compared to the seawater condition. (C) 150 bacterial coastal marine isolates from Nahant, MA, were screened for vitamin auxotrophies in a high-throughput format. Top left, example of growth with and without vitamin mix over three daily transfers, due to auxotrophy for B1. Chitin degraders were prototrophic (marked with circles on tree). Abbreviations: *Vibrio*. = *Vibrionales*; *Altero*. = *Alteromonadales*; *Pseudo*. = *Pseudomonadales*; *Campylo. = Campylobacterales; Rhodo*. = *Rhodobacterales*; *Flavo*. = *Flavobacteriales*.; *fibro. = Fibrobacterales.*

We found that adding vitamins drastically altered the community composition and functional potential of seawater microcosms, indicating that these communities are indeed vitamin limited (Fig. 1). Key taxonomic groups that typically dominate chitin communities, such as *Vibrionales*, *Alteromonadales*, and some *Pseudomonadales,* reached a higher relative abundance in the absence of vitamins (Fig. 1A, Supplementary Fig. S2). This indicates that these taxa may have a competitive advantage in this condition and are likely vitamin producers (termed prototrophs). In vitamin-supplemented samples, taxa including *Rhodobacterales* and *Flavobacteriales* were enriched, suggesting that these may be auxotrophs for one or more vitamins (Fig. 1A). These taxonomic shifts led to differences in functional potential: vitamin-supplemented communities had a lower fraction of chitin degraders, as inferred from the frequency of chitin-hydrolysis protein domains in the metagenomes (Fig. 1B).

### B-vitamin auxotrophies are prevalent in marine particle-associated isolates

To understand the mechanisms driving the community response to vitamins, we characterized vitamin auxotrophies in a collection of strains from the same coastal sampling point, previously isolated on a range of marine polysaccharides, including chitin, chitosan, carageenan, agarose, and alginate (Supplementary Dataset S1) [31]. We characterized 150 seawater isolates representative of five of the orders affected by vitamin supplementation (Fig. 1C). To identify auxotrophies, we grew the isolates in a defined growth medium with and without vitamin supplementation (Table 1). We measured growth over three daily growth-dilution cycles, to dilute vitamins from the initial seed cultures to picomolar concentrations (Table 1) and allow initial intracellular vitamin levels to be diluted as the cells divided [53]. Isolates with reduced growth without vitamins (Supplementary Fig. S1) were then assessed for growth with and without each vitamin individually to identify specific auxotrophies (Fig. 1C, top  left).

Consistent with the seawater microcosm experiment (Fig. 1A), all *Vibrionales* isolates were prototrophs, whereas 87% of the *Rhodobacterales* isolates were auxotrophic for one or more vitamins (Fig. 1C). These results support the hypothesis that the community response to vitamins in the seawater microcosms was likely driven by auxotrophies. Overall, almost one-third of the particle-associated isolates (47/150) were vitamin auxotrophs (Fig. 1C). Further characterization revealed that the auxotrophies were for one or more of five key B vitamins: thiamine (B1), niacin (B3), biotin (B7), folic acid (B9), and cobalamin (B12).

We hypothesized based on the seawater microcosm data that degraders would likely be prototrophs, as chitin-hydrolysis genes were more abundant in the absence of added vitamins. Indeed, in a subset of 65 isolates characterized for growth on chitin [4, 9], the 12 degraders were exclusively prototrophic, with representatives from the clades *Vibrionales*, *Alteromonadales*, and *Pseudomonadales* (Fig. 1C). This was further supported by the isolate genomes, in which both chitin and alginate hydrolysis genes were enriched in prototrophs (Supplementary Fig. S3; Wilcoxon rank sum test: *P*-value < .001). The data suggest that vitamin prototrophy may provide a competitive advantage for degraders in particle-associated communities in the ocean.

### Auxotrophies evolve through partial loss of biosynthesis pathways

**Figure 2:**
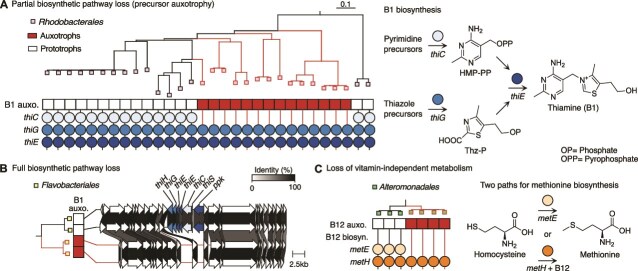
Three evolutionary modes of auxotroph formation. (A) Partial pathway loss leading to formation of B1 pyrimidine auxotrophs in *Rhodobacterales*. Right, three critical genes in the thiamine biosynthesis pathway: *thiC*, which forms the pyrimidine precursor 4-amino-5-hydroxymethyl-2-methylpyrimidine pyrophosphate (HMP-PP); *thiG*, which forms the thiazole precursor 2-(2-carboxy-4-methylthiazol-5-yl) ethyl phosphate (Thz-P); and *thiE*, which couples the two moieties to form thiamine. (B) Total pathway loss. The genomic region of the thiamine biosynthesis cluster is lost in *Flavobacteriales* B1 auxotrophs compared to two closely related prototrophic isolates, with flanking regions conserved. (C) Loss of vitamin-independent metabolism. Both prototrophic and auxotrophic *Alteromonadales* isolates are missing B12 biosynthesis genes and can scavenge B12. B12 auxotrophs have lost *metE*, retaining only the B12-dependent pathway for the biosynthesis of methionine via *metH*.

To establish the genetic basis of vitamin auxotrophies, we compared the results of the phenotype characterization to the isolate genomes. We found a strong match between the observed auxotrophies and the absence of key vitamin biosynthesis genes based on previous studies (Supplementary Fig. S4, Supplementary  Table S1) [15, 16, 36–39]. Based on the presence–absence of these genes, the genomes were assigned as predicted prototrophs or auxotrophs. These predictions were generally accurate for B1, B3, and B7, with error rates between 3% and 7% (Supplementary Table S2). This indicates that the observed auxotrophies were primarily the result of gene loss, as opposed to differential gene regulation or experimental error. In almost all cases, only part of the biosynthetic pathway was lost, resulting in auxotrophies for vitamers to feed into the remaining biosynthetic steps. For example, B1 auxotrophy in *Rhodobacterales* was driven by the loss of a key biosynthetic gene, *thiC*, resulting in an inability to synthesize the pyrimidine subunit of B1, 4-amino-5-hydroxymethyl-2-methylpyrimidine (HMP), although the rest of the pathway was retained (Fig. 2A) [54]. We observed fewer cases in which the entire biosynthetic pathway was lost, for example for B1 in *Flavobacteriales* (Fig. 2B). These results are in agreement with recent studies showing the key role of vitamin precursors and degradation products in cross-feeding [11, 13, 15, 16, 23, 55–57]. Rare auxotrophies were less accurately predicted in this dataset: neither of the only two B9 auxotrophs in the collection were predicted correctly from their genomes.

**Figure 3:**
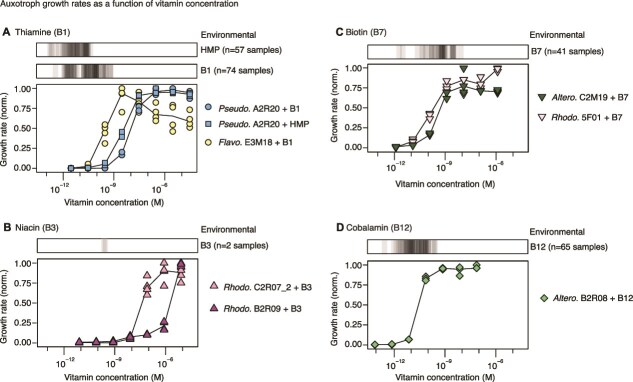
Auxotroph growth rates are limited under environmental vitamin concentrations. Auxotrophs were grown on different vitamin or precursor concentrations: (A) Thiamine (B1) and the B1 vitamer 4-amino-5-hydroxymethyl-2-methylpyrimidine (HMP), (B) Niacin (B3), (C) Biotin (B7), and (D) Cobalamin (B12). Cultures were grown for three growth-dilution cycles, diluting 1:40 every 24 hours. After the last transfer, a kinetic growth measurement was run and growth rates calculated from the exponential phase. Literature values for vitamin concentrations measured in the surface oceans are shown as gray bars above each panel (*n* = 193 samples, Supplementary Dataset S2) [43, 59–85]. Abbreviations: *Altero*. = *Alteromonadales*; *Pseudo*. = *Pseudomonadales*; *Rhodo*. = *Rhodobacterales*; *Flavo*. = *Flavobacteriales*.

In contrast to the accurate predictions for B1, B3, and B7, there was little correlation between vitamin B12 auxotrophies and predictions based on biosynthesis genes (Supplementary Fig. S4, Supplementary Table S2). Vitamin B12 is unique amongst the vitamins discussed here because it is not universally essential. Many bacteria and algae can scavenge and use B12 if it is present but do not have an obligate requirement for it, as they have alternate metabolic pathways that circumvent the B12-dependent enzymes [58]. This describes the majority of particle-associated isolates in this collection: they do not produce B12 but also do not require it, and are therefore not auxotrophs. Methionine biosynthesis is an example of an essential pathway with two alternate isozymes, one B12-dependent (*metH*) and one not (*metE*) (Fig. 2C). We find a small clade of *Alteromondales* B12 auxotrophs that have only *metH* and lack a full B12 biosynthesis pathway, making them reliant on external B12 for methionine biosynthesis (Fig. 2C). In contrast, a neighboring clade of isolates are prototrophs even though they also cannot produce B12 (Fig. 2C). This is possible because they have both *metH* and *metE* and can therefore either scavenge B12 or survive without it (Fig. 2C). Only around 30 isolates in the collection were predicted to produce B12, primarily *Rhodobacterales* isolates that lack *metE* and therefore have obligate B12 requirements (Supplementary Fig. S5). Because the biosynthesis of B12 involves over 30 genes, B12 producers are commonly classified based on percent pathway completeness rather than specific genes [39]. Here, this approach led to misclassifying some auxotrophs as prototrophs, as they lost only single genes and are likely precursor auxotrophs (Supplementary Fig. S5).

Using the genome-based auxotrophy predictors, we expanded our analysis and found that the positive correlation between vitamin prototrophy and polysaccharide degradation is more broadly generalizable. We annotated over 11 000 diverse reference genomes from proGenomes [31] and assigned auxotrophies for vitamins B1, B3, B7, and B9. On average, vitamin prototrophs had a higher number of chitin and alginate lyases, as well as glycoside hydrolases overall, even after normalizing by genome size (Supplementary Figs S6–S7). Genomes with all four vitamin auxotrophies had the fewest glycoside hydrolases on average. This suggests that polysaccharide degraders and vitamin auxotrophs may occupy separate ecological niches across environments.

### Auxotroph growth rates are limited under environmental vitamin concentrations

How do vitamin auxotrophs survive in the open oceans? The concentration of vitamins varies widely in the coastal oceans and is undetectable (sub-picomolar levels) in many areas [59]. Bacterial growth limitation by vitamins in the oceans has been previously shown through mesocosm experiments, for example for B12 [23]. Because vitamins are required in low amounts and can be recycled, it has been proposed that amounts sufficient for survival can be scavenged from seawater [21]. Alternatively, vitamin auxotrophs may require cross-feeding from more direct interactions with vitamin producers. The likelihood of either strategy depends on the amount of vitamins required to sustain growth. To measure vitamin half-saturation constants, the growth of auxotrophs was measured across vitamin concentrations spanning seven orders of magnitude. We compared these values to environmental measurements of dissolved vitamins (*n* = 307 measurements, Dataset S2) [43, 56, 59–85].

**Table 2:** Estimated half-saturation constants (K_S_).

**Order**	**Genus**	**Strain**	**Vitamin**	**K**_**S**_ **(M)**	**Std. error (M)**
*Pseudomonadales*	*Marinobacter*	A2R20	B1	9.6E-09	9.0E-10
			HMP	3.5E-09	2.4E-10
*Flavobacteriales*	*Arenibacter*	E3M18	B1	1.7E-10	5.5E-11
*Rhodobacterales*	*Roseovarius*	B2R09	B3	4.6E-06	1.1E-06
*Rhodobacterales*	*Paracoccus*	C2R07_2	B3	3.7E-08	6.4E-09
*Rhodobacterales*	*Shimia*	5F01	B7	1.3E-10	2.6E-11
*Alteromonadales*	*Colwellia*	C2M19	B7	3.1E-10	6.7E-11
*Alteromonadales*	*Shewanella*	B2R08	B12	2.8E-11	4.6E-12

The half-saturation constants of auxotrophs ranged from ~30 pM for a B12 auxotroph, to almost 5 μM for a B3 auxotroph (Fig. 3, Table 2). This variation over five orders of magnitude mirrors the range of reported intracellular levels for these vitamins, from ~20 molecules per cell (B7) to over 140 000 (B3) [43]. Although the two B7 auxotrophs had similar requirements, there was variation between auxotrophs for both B1 and B3. We hypothesized that this is due to different affinities for vitamins and their precursors, as has been shown for B1 in SAR11 [11]. However, the B1 auxotroph *Pseudo.* A2R20 had similar half-saturation constants for B1 and the B1 vitamer HMP, both an order of magnitude higher than the *Flavo.* E3M18 auxotroph (Fig. 3, top  left). It is possible that this difference stems from variation in transporters or physiology, or that affinities may differ for other vitamers that were not tested here.

The growth rates of all auxotrophs would be limited under the majority of measured environmental vitamin concentrations in seawater (Fig. 3, gray bars; Dataset S2). This was especially stark for vitamin B3, which was recently quantified at ~50 pM in seawater [43, 84], 3–5 orders of magnitude lower than the requirements measured here. Although far fewer measurements exist for particulate fractions, vitamin levels are similar to or even lower in particulate organic matter than in dissolved seawater (Supplementary Dataset S2) [56, 77, 84]. The data confirm that auxotroph growth is likely often vitamin-limited in the global oceans, especially in nutrient-rich particle communities where other resources are at high local concentrations. The striking similarity between the environmental concentrations and the measured bacterial affinities suggests that bacterial uptake may dictate vitamin levels in parts of the oceans, by depleting them to the lowest concentration possible.

### Auxotroph co-cultures are vitamin limited

**Figure 4:**
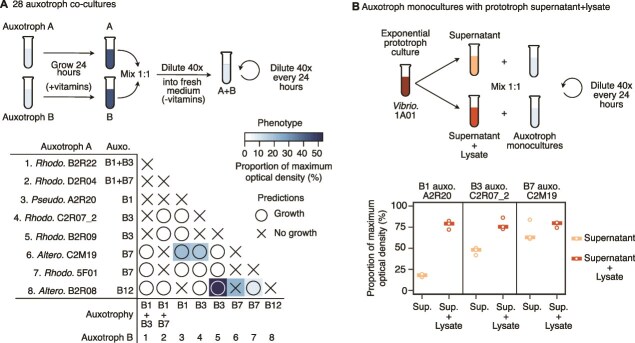
Auxotrophies are only partially alleviated in co-culture and by supernatants. (A) Eight auxotrophs were grown in co-culture, for a total of 28 pairs. Top, the auxotrophs were grown separately with vitamins, then combined 1:1 and transferred to fresh medium without vitamins. Growth was measured after three growth-dilution cycles. Bottom, only 4/17 co-cultures predicted to successfully cross-feed (circles) grew to detectable levels, as well as 1/11 of the co-cultures predicted not to grow. The co-cultures reached between 7% and 52% of the final optical density of co-cultures with vitamin supplementation (% maximum optical density, shading). The 8 monocultures appear on the diagonal. (B) Three auxotrophs (auxotrophs 3, 4, and 6) were further tested for growth with supernatants (left) and lysates containing both supernatant and lysed cells (right), collected from a *Vibrio.* 1A01 prototroph culture in late exponential phase. Growth was measured after 3 growth-dilution cycles. Data are presented as a percentage of the optical density obtained when the supernatant is supplemented by vitamins (% maximum optical density), to correct for the effect of the depletion or release of additional nutrients in each condition (see Fig. S9 for raw data). Abbreviations: *Vibrio*. = *Vibrionales*; *Altero*. = *Alteromonadales*; *Pseudo*. = *Pseudomonadales*; *Rhodo*. = *Rhodobacterales*.

If ambient vitamin levels in seawater are not sufficient for growth, vitamin auxotrophs likely rely on cross-feeding with community members. Mutualistic cross-feeding between auxotrophs has been shown in bacterial–algal interactions, in which bacteria provide B12 and algae provide carbon or other vitamins, including B3, B7, and B9 [17, 86, 87]. Successful complementation in co-culture has been shown with engineered vitamin auxotrophs [88, 89], as well as in natural isolates from this collection, for degraders supporting cross-feeders through organic acids [3, 90]. We therefore hypothesized that particle-associated auxotrophs would complement one another’s vitamin requirements during growth. To test this, we grew a selection of eight auxotrophs in co-culture, including two dual auxotrophs. Each auxotroph was initially grown in monoculture to high density in vitamin-supplemented medium then combined in pairs (Fig. 4A, top). We measured co-culture growth after three growth-dilution cycles, diluting 1:40 every 24 hours, and compared it to growth in vitamin-supplemented medium. Of the 28 co-cultures, 17 pairs were predicted to be able to successfully complement one another’s vitamin auxotrophies, and 11 pairs were predicted not to grow.

Most auxotroph pairs did not grow in co-culture (Fig. 4A). Only around one quarter (4/17) of the predicted successful pairs grew to detectable levels, and that growth was vitamin-limited, ranging from just 7% to 52% of growth with vitamin supplementation. The extinction of most of these co-cultures indicates that they doubled fewer than ~5 times over 24 hours (log_2_ of the dilution factor 40×). This is despite being initially combined in relatively high density after growth in vitamin-rich medium to avoid Allee effects, i.e. limitations due to positive density-dependent growth [91]. Although the two dual auxotrophs did not grow in any co-culture, the other six isolates each grew in at least one instance. Almost all of the 11 co-cultures predicted not to grow indeed did not, except for one: the *Altero.* auxotrophs for B7 (C2M19) and B12 (B2R08). Although C2M19 is unable to synthesize B12 based on its genome, it can produce methionine, which can alleviate the need for B12 in the *Altero.* B12 auxotrophs (Fig. 2C). Overall, even when cross-feeding was theoretically possible, most pairs of auxotrophs were not able to grow together in co-culture. The data indicate that many isolates do not secrete sufficient levels of vitamins during growth to support the growth of other auxotrophs, even under the pressure of mutual extinction.

We hypothesized that prototrophic degraders supply vitamins to auxotrophic cross-feeders (Fig. 4B). Although a subset of auxotrophs grew with prototrophs in co-culture to varying ratios (Supplementary Fig. S8), prototroph growth rates were higher than auxotrophs and quickly reached stationary phase. We therefore could not differentiate between facilitation by secretion of vitamins during growth or by lysis in stationary phase. To further study the mechanism of facilitation, we selected the *Vibrio.* chitin degrader 1A01, which is predicted to produce vitamins B1, B3, and B7, and collected filter-sterilized supernatants and mechanical lysates during late exponential growth (Fig. 4B, top; Supplementary Fig. S9). We grew three auxotrophs in monoculture supplemented with the supernatants, which contain any vitamins secreted during growth, or mechanical lysates, which contain both supernatants and lysed cells and represent the total vitamins available (Fig. 4B, bottom). The supernatants and mechanical lysates were mixed 1:1 with fresh medium with no added vitamins, to ensure that other nutrients were not limiting. All data are presented as a percentage of yield in supernatants with added vitamins, to represent the maximal possible yield given the depletion or release of other nutrients (see Supplementary Fig. S9 for raw data).

Auxotroph growth in monoculture was partially restored by prototroph supernatants and more so by mechanical lysates, depending on the vitamin (Fig. 4B, bottom). All three auxotrophs reached ~78% of maximal yield when grown on mechanical lysates, indicating that total vitamin levels in prototroph cells and supernatants could support high levels of auxotroph growth (Fig. 4B, red). Based on each auxotroph’s yield as a function of vitamin concentration, we estimated the total concentration of vitamers in the supernatants and lysates using a power law fit, finding ~60 nm for B1 vitamers, 100 nm for B3 vitamers, and 2 nm for B7 vitamers (Supplementary Fig. S10, Supplementary Tables S3–S4).

Growth on supernatants alone was variable for the three different vitamin auxotrophs, ranging from only 18% to 69% of maximal yield (Fig. 4B, peach). We estimate that the lysate contained ~5× more B1 vitamers as the supernatant (Supplementary Table S4). For B3 and B7, the reverse was true: the supernatant contained ~2× more vitamers compared to the lysate alone. The extent of facilitation also depended on the physiology of the prototroph, with different trends for each vitamin in early exponential and stationary phase cultures (Supplementary Fig. S9). This indicates that the externalization of vitamins during growth is highly variable even for a single marine isolate and depends on the identity of the vitamin and the growth phase. Based on this data, we conclude that both lysis and secretion are viable mechanisms to alleviate vitamin growth limitations for auxotrophs in co-culture.

### Vitamin cross-feeding alleviates auxotrophies on model particles under diffusion

**Figure 5:**
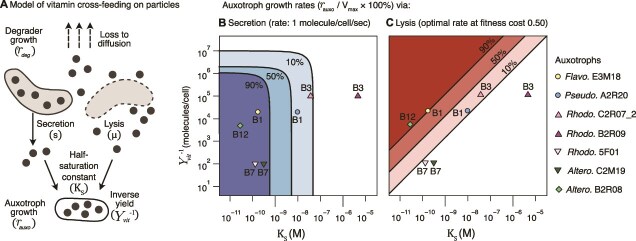
Model of vitamin cross-feeding in a particle-associated community. (A) Scheme of model particle community. Degraders supply vitamins to auxotrophs through secretion (s) or lysis (μ). The concentration of substrates and vitamins depends on the rate of diffusion and the population size of degraders, based on their effective growth rate (*r_deg_*). The auxotroph growth rate (*r_auxo_*) depends on cellular vitamin requirements, i.e. inverse yield (molecules/cell; Y_vit_^−1^), and uptake efficiency, i.e. half-saturation constants (K_S_). (B and C) Auxotroph growth rates via secretion (left) and via lysis (right) is shown in shaded areas (>90% of maximal growth rate, dark; >50%, medium; >10%, light). Values for measured K_S_ values and estimated vitamin yields for auxotrophs are marked by symbols. Growth under secretion (B) is characterized by two threshold values: a vertical line corresponding to the maximal half-saturation constant, and a horizontal line corresponding to the maximal yield. Growth under lysis (C) is approximately proportional to the product of the inverse vitamin yield and half-saturation constant. Abbreviations: *Altero*. = *Alteromonadales*; *Pseudo*. = *Pseudomonadales*; *Rhodo*. = *Rhodobacterales*; *Flavo*. = *Flavobacteriales*.

In the open oceans, metabolites are rapidly lost to diffusion. Under these conditions, cross-feeding requires high metabolite concentrations and/or high uptake affinities, even in densely packed and spatially structured particle communities. Given these constraints, we asked whether vitamin cross-feeding can be sustained on particles through lysis or secretion without leading to community collapse. To this end, we developed and parameterized a spatial model for the growth of degraders and auxotrophs on a particle, accounting explicitly for the production, diffusion, and uptake of substrates and vitamins (Fig. 5A, Supplementary Figs S11–S12, Supplementary Tables S5–S6; model details in SI text).

Both degrader lysis and secretion can sustainably support growth on particles under diffusion for the majority of auxotrophs in this work, according to the model predictions (Fig. 5B and C). Auxotroph growth rate depends on cellular vitamin requirements, defined by two parameters: the inverse yield (Y_vit_^−1^), which sets the required intracellular vitamin concentrations in growing cells, and the uptake efficiency, i.e. the half-saturation constants to vitamins (K_S_). Depending on whether vitamins are secreted or released by lysis, our model predicts qualitatively different dependencies between prototroph and auxotroph populations.

Auxotroph growth rates under secretion are characterized by two threshold values. First, the maximal half-saturation constant for a given growth rate, which represents the minimal affinity needed for growth on the low vitamin concentrations maintained by diffusion. Second, a maximal inverse yield for a given growth rate, at which point the rate of vitamin consumption by auxotrophic cells equals the rate of vitamin production by prototrophs (Fig. 5B). Secretion will alleviate vitamin limitation more easily for auxotrophs with better affinities (i.e. lower K_S_) and lower vitamin requirements (Y_vit_^−1^), allowing them to grow faster. Higher secretion rates will enable higher auxotroph growth rates if vitamin secretion is costless for prototroph cells. However, if secretion is costly, an intermediate secretion rate will be optimum for auxotroph growth, because it balances the trade-off between the number of vitamins produced per cell and prototroph growth rates, to maximize the vitamins secreted by the population as a whole (Supplementary Fig. S12).

In contrast to secretion, auxotroph growth under lysis is approximately proportional to 1/(K_S_  ^*^ Y_vit_) (Fig. 5C). This is because lysis couples the vitamin concentration inside the prototroph cell with the number of vitamins they release. Therefore, higher inverse yields result in more vitamin availability and higher auxotroph growth rates. Similar to a costly secretion scenario, auxotroph growth reaches an optimum at an intermediate lysis rate, equivalent to half of the maximal prototroph growth rate (i.e. a fitness cost of 50%) (Supplementary Fig. S12). Below this optimum, increasing lysis results in increased auxotroph growth, as more vitamins are released. However, as lysis rates increase past the optimum, the prototroph population approaches a collapse, which reduces the growth rate of auxotrophs.

We find a positive correlation between the half-saturation constants measured in this work and the estimated inverse yields (ρ = .64), leading these points to appear approximately along the diagonal (Fig. 5B and C). This suggests some level of evolutionary fine-tuning for affinities that match vitamin requirements: higher affinities evolve to efficiently consume rare resources, and lower affinities for more abundant resources. Auxotroph growth rates span a wide range of values for both lysis and secretion, suggesting that auxotroph growth is sensitive to variations in vitamin yields, affinities, and local lysis or secretion rates.

Although we find that auxotroph growth from both mechanisms is generally possible, cross-feeding via secretion is more effective for vitamins with low cellular requirements, and lysis for vitamins highly abundant in cells. For example, the two B7 auxotrophs, which have high affinities and low cellular requirements, grow at over 90% of their maximal growth rate via secretion and under 10% via lysis (Fig. 5B and C). However, this is highly dependent on the estimated number of molecules of B7 per cell, which changes drastically based on which vitamer is considered [16]. The B3 auxotroph *Rhodo.* B2R09 was predicted to grow extremely slowly in both scenarios (Fig. 5B and C), due to its exceptionally poor affinity for the B3 vitamer tested here (nicotinic acid). Other mechanisms may explain the growth of this auxotroph in nature, including a lower K_S_ for other vitamers not tested here.

## Discussion

We found that B vitamin dependencies are widespread in marine particle-associated communities. Even in nutrient-rich coastal seawater microcosms, vitamins were limiting and altered particle community assembly and function (Fig. 1). We studied community interactions in detail through large-scale characterization of 150 naturally co-existing, environmentally relevant, and taxonomically diverse bacterial isolates. Almost a third of particle-associated isolates were vitamin auxotrophs (Fig. 1), consistent with estimates of widespread auxotrophies based on marine bacterial genomes [12, 87] (Fig. 2). We report that vitamin auxotrophies mapped to trophic community structure and are enriched in cross-feeders, in contrast to prototrophic chitin degraders (Fig. 1). We experimentally measured vitamin affinities for copiotrophic isolates across multiple auxotrophies, showing that these auxotrophs are vitamin limited in much of the surface oceans (Fig. 3). Through co-culture experiments, we showed that vitamin secretion is highly condition-dependent (Fig. 4), indicating that vitamin cross-feeding may proceed through both lysis and secretion in particle-associated communities. Based on our experimental measurements, we modeled growth on particles and found that auxotrophs can stably persist in communities through both lysis and secretion, even under conditions of maximal diffusion (Fig. 5).

Vitamin dependencies map to the trophic structure of degraders and cross-feeders in particle-associated communities [4, 90]. We found that vitamin auxotrophies were enriched in generalist cross-feeders in this collection (Fig. 1C). This was in contrast to the chitin degraders, which were exclusively prototrophs. This was further supported by the seawater microcosm data, in which chitinase genes were more abundant in samples without vitamin supplementation (Fig. 1B). Thus, in particle communities, degraders may provide both carbon-rich substrates and vitamins to auxotrophic cross-feeders in a one-sided interaction, in contrast to the two-way, mutual symbiosis in algal–bacterial interactions [17]. Previous studies on succession in particle communities have shown that degraders are the first to arrive on particles, followed by a wave of cross-feeders [4]. It may therefore be more advantageous for pioneering degraders to be self-sufficient prototrophs than for cross-feeders, which are reliant on other community members regardless. We observed the same pattern in a broad analysis of vitamin auxotrophies and polysaccharide degradation genes in over 11 000 genomes (Supplementary Figs S6–S7), indicating that this division into separate ecological niches may be more broadly applicable and merits further study.

This study focuses primarily on chitinous particles and on microbial communities from a coastal sampling point in the western Atlantic Ocean (Nahant, MA, USA). Bacterial community dynamics are likely to differ in the context of other types of marine snow particles rich in other polysaccharides or proteins [92]. It may also differ during a phytoplankton bloom due to the production of vitamins by algae, such as B1 and B7 [17, 75, 86, 87]. Simplified model systems like the chitin particles used here do not reflect the compositional and structural complexity of natural particles [92]. The colonization of chitin particles is also difficult to study with individual isolates under controlled conditions, as the attachment depends on flow and myriad other factors. Despite these limitations, a recent study showed evidence for successional dynamics on natural marine snow, similar to what has been observed in simplified models [93]. Additionally, the seawater microcosm experiment was performed using seawater collected on a single sampling day, providing a snapshot of a dynamic and rapidly changing ecosystem. A previous time series study at the same sampling location showed that the microbial community undergoes frequent and rapid shifts in composition, on the order of days or weeks [94]. These changes in community composition are likely to impact the prevalence of vitamin auxotrophies as well and should be explored in future studies.

The experimental verification of auxotrophies in this study support previous estimates that vitamin auxotrophy is widespread amongst marine bacteria [12]. Our results generally confirm the accuracy of genomic predictors for auxotrophies, especially for vitamins B1, B3, and B7 [15, 16, 36–39]. In the majority of cases, only part of the biosynthetic pathway was lost, as has been recently observed in other vitamin auxotrophy studies in marine bacteria [11, 13, 15, 16, 23, 55, 56]. This partial pathway loss leads to auxotrophies for vitamers, including precursors and degradation products, and may also make it possible to regain vitamin biosynthesis through horizontal gene transfer. Indeed, previous studies have found vitamin-related genes on mobile genetic elements [95, 96], including for the thiamine biosynthesis gene *thiC* in a marine *Roseobacter* [97]. The patchy distribution of auxotrophies suggests that vitamin biosynthesis genes may be frequently lost in particle-associated communities in line with the Black Queen Hypothesis [98]. Comparative genomics studies are needed to determine whether auxotrophies evolved multiple times through independent loss events, as has been shown in algae [99] and suggested for vitamin B12 in soil bacteria [100].

We estimate the overall concentration of vitamers in the supernatants and lysates of a vitamin-producing strain based on the extent of auxotroph growth facilitated (Supplementary Fig. S9, Supplementary Tables S3–S4). These measurements are based on vitamer usage and therefore encompass any vitamer form utilized by auxotrophs. Thus, they are complementary to previously published mass spectrometry measurements, as growth-based measurements are truly untargeted and are not limited by *a priori* knowledge of relevant vitamer compounds. There is an emerging consensus that vitamers, such as vitamin precursors and degradation products, play key roles in vitamin cross-feeding, including for B1 [11, 13, 15, 56] and B12 [23, 55, 88, 101], and as recently shown for B7 [16]. The concentration of a vitamer can exceed vitamin levels by orders of magnitude, for example the B7 precursor desthiobiotin in *Vibrio campbellii* [16]. Recent advances in mass spectrometry have made it possible to quantify trace levels of vitamins and vitamers in both cultures and environmental samples [43, 56, 85]. Future work is needed to analyze the vitamers produced by a wider range of natural isolates and determine their relative contributions to cross-feeding. Additionally, more environmental measurements of particulate vitamins and vitamers are needed to understand particle community dynamics: only 3 out of 28 marine vitamin quantification studies cited here analyzed the particle-associated fraction alongside the dissolved [56, 77, 84].

Whereas previous work on vitamins in marine bacteria has primarily focused on vitamins B1, B7, and B12, our results highlight gaps in research on vitamins B3 and B9. We find that B3 auxotrophs are prevalent in these communities and had the highest vitamin requirements of any of the isolates tested, in keeping with high cellular levels of nicotinamide adenine dinucleotide (NAD+), the product of B3. Future studies are needed to better understand how these auxotrophs are meeting their vitamin requirements in the environment to produce sufficient amounts of this crucial redox-active metabolite. Additionally, B9 auxotrophy was rare in this collection and was difficult to predict from the genomes, resulting in both false positives and false negatives. This suggests that there may be additional genes or pathways for B9 biosynthesis that may be discovered in future studies of larger numbers of auxotrophs.

The prevalence of auxotrophies in particle communities indicates that even under the harsh conditions of diffusion in the open oceans, auxotrophs are able to scavenge sufficient vitamins to survive. We modeled cross-feeding on particles under diffusion and found that in most cases a relatively low rate of vitamin release through lysis or secretion is sufficient to alleviate vitamin limitation (Fig. 5). Vitamin affinities are also negatively correlated with vitamin requirements, suggesting that like prices in a market, affinities are higher the rarer the commodity. In general, we found that cross-feeding via secretion is more effective for vitamins with low cellular requirements, and lysis for vitamins highly abundant in cells. Our results suggest that auxotroph growth on particles exists on a tight margin, sensitive to small changes in affinities, yields, and lysis or secretion rates.

Vitamin cross-feeding can take place through a number of mechanisms, including externalization during growth through secretion or leakage, and release from dying cells [102, 103]. Previous studies in particle-associated communities have focused on public goods shared through secretion or leakage, including sugars released from polysaccharide degradation [9], excreted organic acids [90], and secreted siderophores [8]. However, there is also an emerging perspective pointing toward lysis and antagonism as a key facilitator of cross-feeding in natural communities [55, 104, 105]. Our results indicate that both secretion and lysis likely mediate vitamin cross-feeding in particle-associated communities. We find that secretion is highly variable and depends on the vitamin, strain, and growth stage, as has been recently shown for B12 produced by marine *Rhodobacterales* species [44]. More studies are needed to better understand the mechanisms by which vitamins are externalized from cells, the fitness costs of vitamin loss during growth, and the prevalence of different externalization mechanisms in the environment.

The relative contributions of lysis and secretion have potential to greatly impact the flow of resources in natural microbial communities. Cross-feeding by lysis is a general mechanism for metabolite exchange, agnostic to species identity. Therefore, lysis-based interactions do not fit as neatly into the paradigm of specific species-species interaction networks. In contrast, secretion seems to be highly dependent on the identity and physiology of the producer. The balance between secretion and lysis is thus key to understanding to what extent microbial community function is shaped by deterministic or stochastic processes.

## Supplementary Material

ISMEJ-Gregor-SI_wraf184

Dataset_S1_wraf184

Dataset_S2_wraf184

## Data Availability

Metagenomic reads from seawater microcosms are accessible through NCBI BioProject (accession number PRJNA1028615). Code used to generate the particle model and data analysis is https://github.com/RachelGregor/marine-vitamin-auxotrophies.
