# Supplementary material for: Vitamin auxotrophies shape microbial community assembly on model marine particles
Source: ISME J. 2025 Aug 20;19(1):wraf184. doi: 10.1093/ismejo/wraf184 (PMC12448291; doi:10.1093/ismejo/wraf184)
Supplement: ISMEJ-Gregor-SI_wraf184 [file ismej-gregor-si_wraf184.pdf]

Vitamin auxotrophies shape microbial community assembly on model marine particles

Rachel Gregor[1], Gabriel T. Vercelli[1], Rachel E. Szabo[1], Matti Gralka[2], Ryan C. Reynolds[3], Evan B. Qu[1], Naomi M. Levine[3], Otto X. Cordero[1]*

[1]Department of Civil and Environmental Engineering, Massachusetts Institute of Technology, Cambridge, MA, 02139-4307, United States

[2]Systems Biology Group, Amsterdam Institute for Life and Environment (A-LIFE) and Amsterdam Institute of Molecular and Life Sciences (AIMMS), Vrije Universiteit Amsterdam, 1081 HZ, Amsterdam, The Netherlands

[3]Department of Biological Sciences, University of Southern California, Los Angeles, CA, 90089-0371, United States

*Corresponding author. Parsons Laboratory, Building 48-428, Department of Civil and Environmental Engineering, Massachusetts Institute of Technology, 15 Vassar St, Cambridge, MA 02139-4307, United States. E-mail: ottox@mit.edu

This PDF file includes:

Supporting text: model of vitamin limitation on particles
Supporting text: calculation of secretion rates
Figures S1 to S12
Tables S1 to S6

Other supporting materials for this manuscript include the following:

Datasets S1 to S2

Supporting Information Text

I: Model of vitamin limitation on particles

To put the measured vitamin auxotrophies in an ecological context, we develop a simplified model of chitin particle degradation in the ocean. The focus of the model is to understand to what extent vitamin release by prototrophic degrader strains can support the growth of auxotrophic cross-feeders. In the following sections, we will first describe the biological parameters in the model controlling degrader and cross-feeder growth. Then, we will move on to the physical parameters describing the geometry of the chitin particle and the spatial dynamics of vitamins and cross-fed metabolites. Lastly, we will provide details about the numerical simulations performed and develop approximate analytical solutions to model equations. We assume the timescale of diffusion processes is much faster than the timescale of microbial growth. Therefore, we use the steady-state solution for all diffusion processes described to calculate biological parameters like growth rates.

Biological parameters. We model cell growth on the chitin particle as a logistic equation and incorporate two possible vitamin release mechanisms: lysis and secretion. The former is described by a single parameter μ, the lysis rate of the population, that multiplied by the population density, σ, constitutes the rate of cell death. The latter is described by two parameters s and c that represent the vitamin secretion rate in $molecules/h$ units and the growth delay per vitamin molecule secreted in units of $h/molecule$, respectively. Assuming each secreted vitamin molecule induces the same growth delay c, the doubling time of a population secreting vitamins at a rate s would obey the following equation:

$$t_{1/2}^{s} = t_{1/2}^{0} + c \cdot s \cdot t_{1/2}^{s}$$

Where $t_{1/2}^{0}$ is the doubling time of the population without any secretion. Isolating $t_{1/2}^{s}$ and remembering that the doubling time is related to the growth rate by the following identity $t_{1/2}^{s} = \frac{\ln(2)}{r_s}$, we arrive at:

$$t_{1/2}^{s} = \frac{t_{1/2}^{0}}{1 - cs} \Rightarrow r_s = (1 - cs)r_0$$

Therefore, the quantity $f = c \cdot s$ is effectively a fitness cost for population growth.

Additionally, releasing vitamins can affect the carrying capacity of the system, k, either directly by wasting resources that would usually be used for growth or indirectly through its effect on the growth rate preventing cells to capture available resources fast enough. To model these effects, we look at metabolite fluxes. We define the total flux of metabolites available to the cell as f_{in} and the amount of this flux used towards biomass as f_b. The difference between f_{in} and f_b is the amount of resource flux either wasted in the form of secreted vitamins or unable to be captured by the cell. Assuming the total number of metabolites M_0 needed for cell division is constant and that the carrying capacity of the system is proportional to the fraction of metabolite flux directed to biomass, we can write the following equations:

$$\frac{M_0}{f_{in}} = t_{1/2}^0 \cdot \frac{f_b^0}{f_{in}} = t_{1/2}^s \cdot \frac{f_b^s}{f_{in}} \Rightarrow k_s = \frac{t_{1/2}^0}{t_{1/2}^s} k_0 = (1 - cs)k_0$$

Therefore, vitamin secretion affects the carrying capacity in the same proportion that it affects the population growth rate.

Condensing all these results, we use the following equation to model microbial growth in the presence of vitamin secretion and cell lysis:

$$\frac{d\sigma}{dt} = r(1-f)\sigma\left(1 - \frac{\sigma}{k(1-f)}\right) - \mu\sigma$$

Where r represents the maximum growth rate of the degrader cells. For the purposes of our model, we assume the degrader population to be at steady-state and therefore not changing with time. Calculating this steady-state density, we arrive at:

$$\sigma^* = \left(1 - \frac{\mu}{r(1-f)}\right) k(1-f)$$

We parametrize this equation with previously measured values for carrying capacity and growth rate for the 1A01 strain, a model chitin degrader [1]. For this strain, the growth rate on chitin particles has been measured to be $0.26\ h^{-1}$ and we estimate the carrying capacity of chitin particles to be about $7\ cells/um^2$ from optical density data collected during the stationary growth phase of 1A01 liquid

cultures [1]. Adopting these numbers, the steady-state density of degraders on the particle becomes only a function of the death rate and secretion fitness cost, going to zero as the death rate approaches the degrader growth rate or the secretion fitness cost approaches one.

Having established the steady-state dynamics of degrader growth and death on the particle, we now consider the ability of cross-feeders to grow in this environment. We assume that the growth of auxotrophic cross-feeders depends on the release of both substrates (for example metabolic byproducts like acetate) and vitamins by degraders. We model the cross-feeder growth rate as a multiplicative Monod function of the concentration of available substrates and vitamins:

$$r_{auxo} = r_{cf}\left(\frac{[S]}{[S] + K_{m,s}}\right)\left(\frac{[V]}{[V] + K_{m,v}}\right)$$

Where S represents the concentration of substrates, V represents the concentration of vitamins, and $K_{m,s}, K_{m,v}$ represent their respective Monod constants. In addition, r_{cf} represents the maximum growth rate of the cross-feeder strain when neither substrates nor vitamins are limiting. We further define the maximum growth rate of the cross-feeder strain for a given substrate concentration:

$$r_{max} = r_{cf}\left(\frac{[S]}{[S] + K_{m,s}}\right)$$

We parametrize these equations with literature values and quantities measured in this study. We take the value for the maximum growth rate of cross-feeders to be $0.3\ h^{-1}$ as estimated in a large screen we previously conducted with the same isolate collection used in this study [2]. We focus on acetate, which is secreted by 1A01 during growth and has been previously shown to support cross-feeding [3]. We use a value of $K_{m,s} = 1\ mM$ based on previous studies of the growth of *E. coli* on acetate [4–6]. We also ran simulations for lower $K_{m,s}$ values for acetate, down to $1uM$, and the results don't change significantly (Fig. S11). For vitamins, we use the half-saturation constants estimated in this study.

Physical parameters. For this simplified model, we assume a radially symmetric, spherical chitin particle with radius $R_0 = 150\ \mu m$. Both degraders and cross-feeders colonize the particle surface. As

the degraders break down the particle and grow, they produce substrates and vitamins that are consumed by the cross-feeders. However, these molecules simultaneously freely diffuse from the surface of the particle into the ocean while they are being consumed, and we model these processes with the following equations:

$$\frac{\partial S}{\partial t} = D_S \nabla^2 S - r_{auxo} \frac{\rho}{Y_S}$$

$$\frac{\partial V}{\partial t} = D_V \nabla^2 V - r_{auxo} \frac{\rho}{Y_V}$$

Where S and V represents the concentrations of metabolic byproducts and vitamins, respectively, D_S and D_V their diffusion constants, and Y_S and Y_V the yield of cells on those resources. Additionally, r_{auxo} is the auxotroph growth rate defined previously and ρ is the volume density of cross-feeder cells. Focusing on the initial moments of cross-feeder colonization of the particle, we assume ρ to be a $1\mu m$ thick monolayer of cells and set the cross-feeder density to 0 beyond that point.

To fully define these equations, we are missing boundary conditions at the surface of the particle and at infinity. For the purposes of our model, we assume that the ocean contains no substrates or vitamins, making their concentration approach zero as we move away from the particle. At the surface of the particle, however, there is a flux of both substrates and vitamins determined by the growth and death rates of degrader cells.

To calculate the flux of substrates, we first calculate the rate of chitin degradation and the release of its monomer, N-acetyl glucosamine (GlcNAc) as the product of the cell density on the particle surface ($\sigma *$), the conversion factor between chitinase mass and cellular biomass (${Y_e}^{-1}$), and the chitinase specific activity (k_e). All these values were experimentally determined for 1A01 previously [1]. We further make the simplifying assumption suggested previously that 1A01 is capable of uptaking all the GlcNAc produced during particle degradation, making the GlcNAc production rate equal to the GlcNAc consumption rate at the particle surface [1]. Finally, we multiply this consumption rate by how many moles of substrates are produced by 1A01 for each mole of GlcNAc consumed, arriving at the rate of substrate production on the particle surface. As an approximation for this metabolic conversion

factor, we consider the ratio of moles of acetate produced to moles of GlcNAc consumed by the degrader ($f_{Act/Gln}$), which was estimated in a recently developed flux balance analysis model for 1A01 [7]. Overall, the final formula for the flux of byproducts on the particle surface becomes:

$$F_M = \frac{\sigma^*}{Y_e} k_e f_{Act/Gln}$$

To calculate the flux of vitamins due to lysis, we simply multiply the cell density on the particle surface (σ^*), the number of vitamins per cell (${Y_V}^{-1}$), and the death rate (μ). And add to that the flux of vitamins due to secretion, calculated as the product of the cell density on the particle surface (σ^*) and the secretion rate (s). The final equation for this flux then becomes:

$$F_V = \frac{\sigma^*}{Y_V} \mu + \sigma^* s$$

The values used for each of these numerical parameters can be found in Table S5.

Numerical simulations. We simulate these equations with the parameters from Table S5 using the py-pde package v0.32.1 in Python v5.3.3. With this package, we create an extension of their standard PDEBase code to include the reaction-diffusion terms shown before. For the simulation space, we use a spherically symmetric grid, no flux boundary conditions on the particle surface, and the following Robin boundary conditions at the opposite end of the simulation grid to account for finite dimensions:

$$\partial f(R_f) + \frac{1}{R_f} f(R_f) = \frac{f(\infty)}{R_f}$$

Where $f(R)$ represents the concentration of substrates or vitamins at a certain distance R from the particle, R_f is the distance from the center of the particle to the outside boundary of the simulation and $f(\infty)$ is the concentration of substrates or vitamins at infinity. This identity can be derived because there are no sources or sinks outside of the simulation grid, so the diffusion-reaction equations can be simplified and analytically solved in that region to be of the form:

$$f(R) = A - \frac{B}{R} \text{ for } R > R_f$$

Where A and B are numerical constants and A equals $f(\infty)$. Applying this analytical formula at the boundary of the simulation, we can calculate the value of f and its derivative at $r = R_f$:

$$f(R_f) = A - \frac{B}{R_f}, \frac{\partial f}{\partial R}|_{R_f} = \frac{B}{R_f^2}$$

Plugging these values back into the expression for our Robin boundary condition verifies its validity. In our simulations, we set $R_f = 160\ \mu m$, making our simulation grid $10\ \mu m$ wide, and $f(\infty) = 0$ since we assume no vitamins or substrates are present in the ocean. We used a spatial discretization length of $0.33\ \mu m$ yielding 30 discrete simulation points.

To numerically estimate the steady state profiles of substrates and vitamins, we initialized their spatial distributions with analytical solutions to the standard diffusion equations obtained by disregarding consumption terms. We then simulated the temporal dynamics of the full reaction-diffusion equation for a period of time 100 times longer than it would take for diffusion alone to homogenize the length of our simulation space:

$$t_{sim} = 100 \cdot t_{diff} = 100 \cdot \frac{1}{2}\frac{L^2}{D}$$

Where $L = R_f - R_0 = 10\ \mu m$ is the length of the simulation grid and $D = min(D_M, D_V)$ is the minimum between the vitamin and substrate diffusion constants. We verified that longer simulation times and initialization of the profiles with the concentrations set to zero everywhere did not change the results of these numerical estimates.

Analytical approximations. In addition to the numerical simulations described previously, we can derive approximate analytical expressions to gain more insight into how each factor in the model contributes to the final results. The main approximation we make is that the non-linear consumption term in the reaction-diffusion equation for vitamins is negligible. This is a reasonable approximation in our case because consumption of vitamins only occurs very close to the surface of the particle and at a

small rate compared to the rate of loss to diffusion. We also numerically confirmed the validity of this approximation for each of the strains for which affinities were measured. This way, we assume the dynamics of vitamins is dominated by diffusion and solve for the vitamin concentration profile (V) as a function of the radial distance from the particle (R).

$$V(R) = \frac{{R_0}^2 F_V}{D_V R}$$

Where, as defined previously, R_0 is the radius of the chitin particle, $\mathrm{F_V}$ is the flux of vitamins at the particle surface, and D_V is the diffusion constant for vitamins.

From this analytical profile, we can calculate the vitamin concentration at the particle surface to be $V(R_0) = \frac{R_0 \mathrm{F_V}}{D_V}$ and get an expression for the growth rate of auxotrophic cross-feeders:

$$r_{auxo} = r_{max}\left(\frac{V(R_0)}{V(R_0) + K_{m,v}}\right) = r_{max}\left(\frac{V(R_0)/K_{m,v}}{V(R_0)/K_{m,v} + 1}\right)$$

Looking at this expression, it becomes clear that we can define an index I that determines the qualitative outcome of growth as follows:

$$I = \frac{V(R_0)}{K_{m,v}}$$

If $I \gg 1$, the growth rate is high, and if $I \ll 1$, it is low.

In cases where lysis is the only vitamin release mechanism, we know $F_V = \frac{\sigma^* \mu}{Y_V}$ and therefore can write

$$I = \frac{R_0 \sigma^* \mu}{D_V Y_V K_{m,v}} \propto \ {Y_v}^{-1} {K_{m,v}}^{-1}$$

The calculation of this quantity for each of the strains for which affinities were measured are summarized in Table S6 (assuming an optimal death rate of $0.13\ h^{-1}$).

In cases where secretion is the only vitamin release mechanism, we know $F_V = \sigma^* s$ and therefore can write:

$$I = \frac{R_0 \sigma^* s}{D_V K_{m,v}} \propto \ {K_{m,v}}^{-1}$$

Therefore, this quantity becomes independent of Y_v, explaining why the 50% growth contour lines in our simulations (equivalent to having I=1 analytically) are nearly vertical in a $K_{m,v}$ vs. Y_v graph for low vitamin yield values. The vitamin yield only becomes an important factor in these calculations when it is so large that the number of vitamins consumed by the cross-feeder cells approaches the number of vitamins secreted by the degrader cells. In this situation, our analytical approximation that vitamin consumption is negligible breaks down and it explains why for high yield values, the 50% growth contour lines in our simulations (equivalent to having I=1 analytically) are nearly horizontal in a $K_{m,v}$ vs. Y_v graph.

II: Calculation of secretion rates

Vitamin secretion rates were estimated as following:

Assuming cells secrete vitamins at a constant rate, s, per cell throughout growth and that all vitamins accumulate in the medium, the number of vitamins present in the medium at any given time, $V(t)$, follows the equation

$$\frac{dV}{dt} = sN(t)$$

Where $N(t)$ is the cell abundance.

We further assume cells were maintained at exponential growth throughout the entire experiment at an exponential growth rate r. This way, we can write $N(t) = N_0 e^{rt}$ and substitute this into the previous equation to arrive at

$$\frac{dV}{dt} = sN_0 e^{rt} \Rightarrow V(t) - V(0) = \frac{sN_0}{r}(e^{rt} - 1)$$

We can also assume that $V(0) = 0$ since no vitamins were present in our experimental medium.

Finally, by looking at the culture at a time t_f, we can estimate s using the following equation

$$s = \frac{V(t_f)r}{N(t_f) - N_0} \approx \frac{V(t_f)r}{N(t_f)}$$

Where we substituted $N(t_f) = N_0 e^{rt_f}$ and used the approximation $N(t_f) \gg N_0$, valid when the concentration of the inoculum in the medium is much lower than the final concentration of cells.

For our numerical calculations, we estimated r through exponential fits of optical density measurements of the experimental cultures (0.00618 1/min), we estimated $V(t_f)$ through power law fits of yield curves for each vitamin (Table S3-S4), and we measured $N(t_f)$ in optical density units (OD 600=0.254).

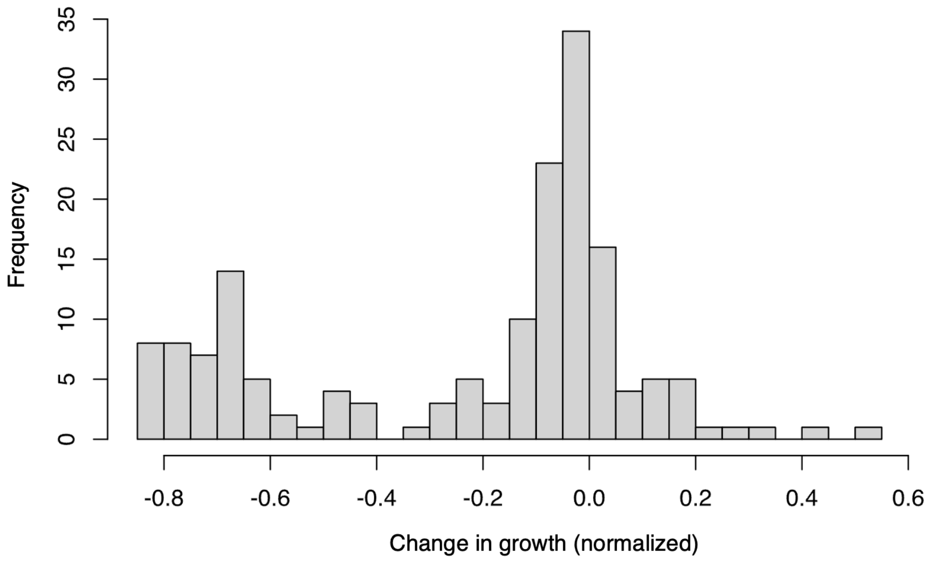

Fig. S1. Change in growth with and without vitamins. The median OD value was calculated for both conditions and the growth deficit calculated as follows: ([MBL-V] – [MBL+V])/([MBL-V] + [MBL+V]). A growth deficit value of -0.4 was chosen as the cutoff for putative auxotrophs based on the distribution of values shown here.

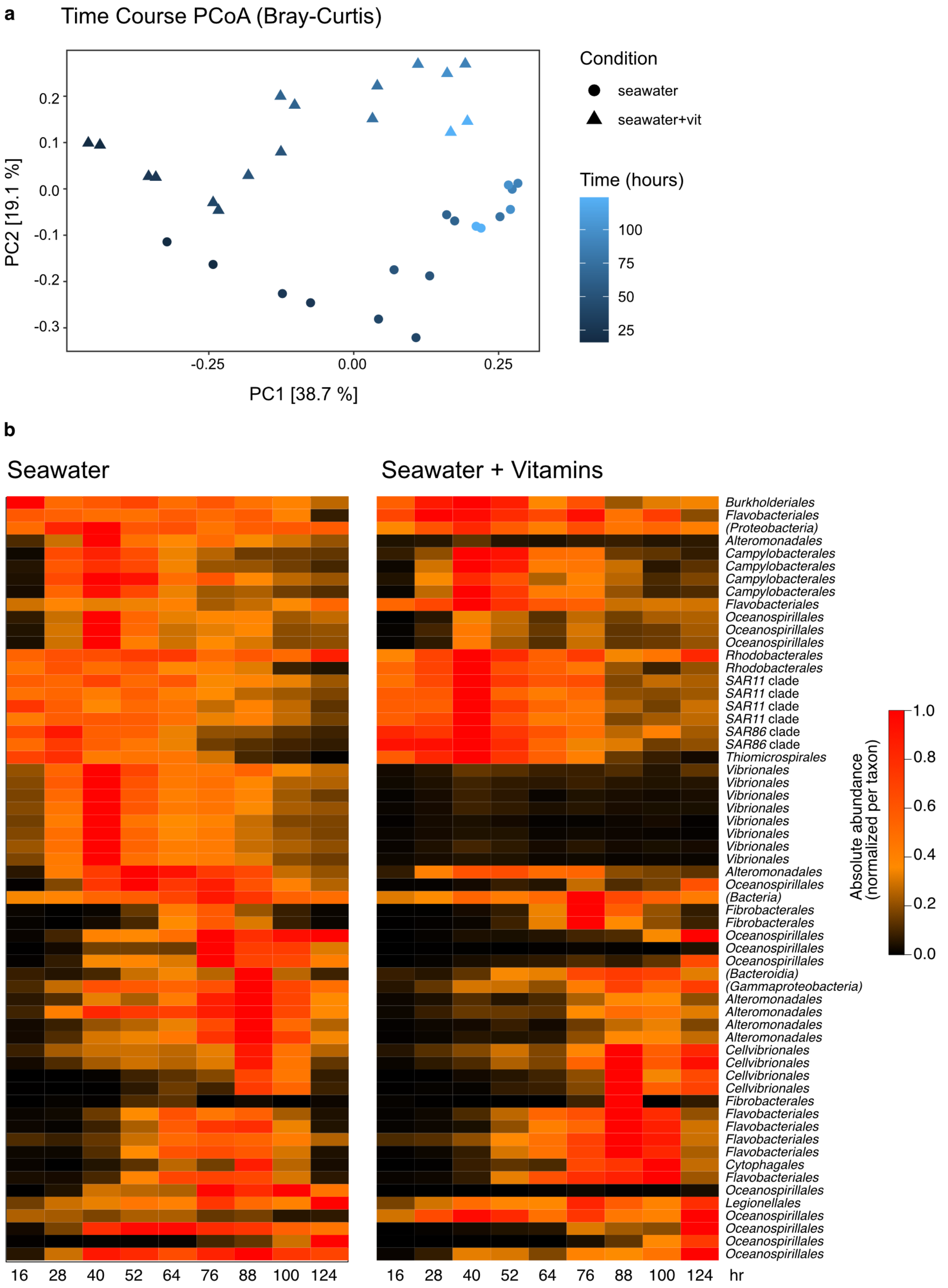

Fig. S2. Taxonomic shifts over time in seawater microcosms with and without added vitamins. (A) The two conditions are distinct over time and (B) differ in the abundance of key taxa. Taxonomy was assigned using PhyloFlash (see Methods).

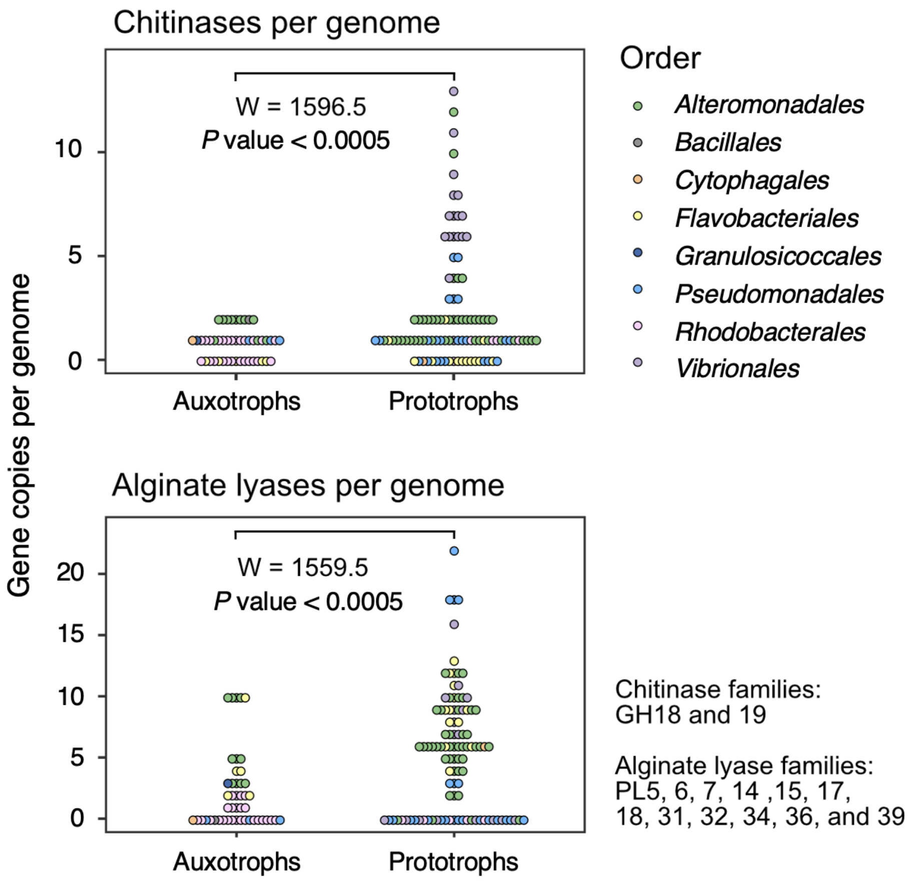

Fig. S3. Chitinases and alginate lyases per genome. Both chitin (top) and alginate (bottom) hydrolysis genes are enriched in prototrophs compared to auxotrophs (Wilcoxon rank sum test).

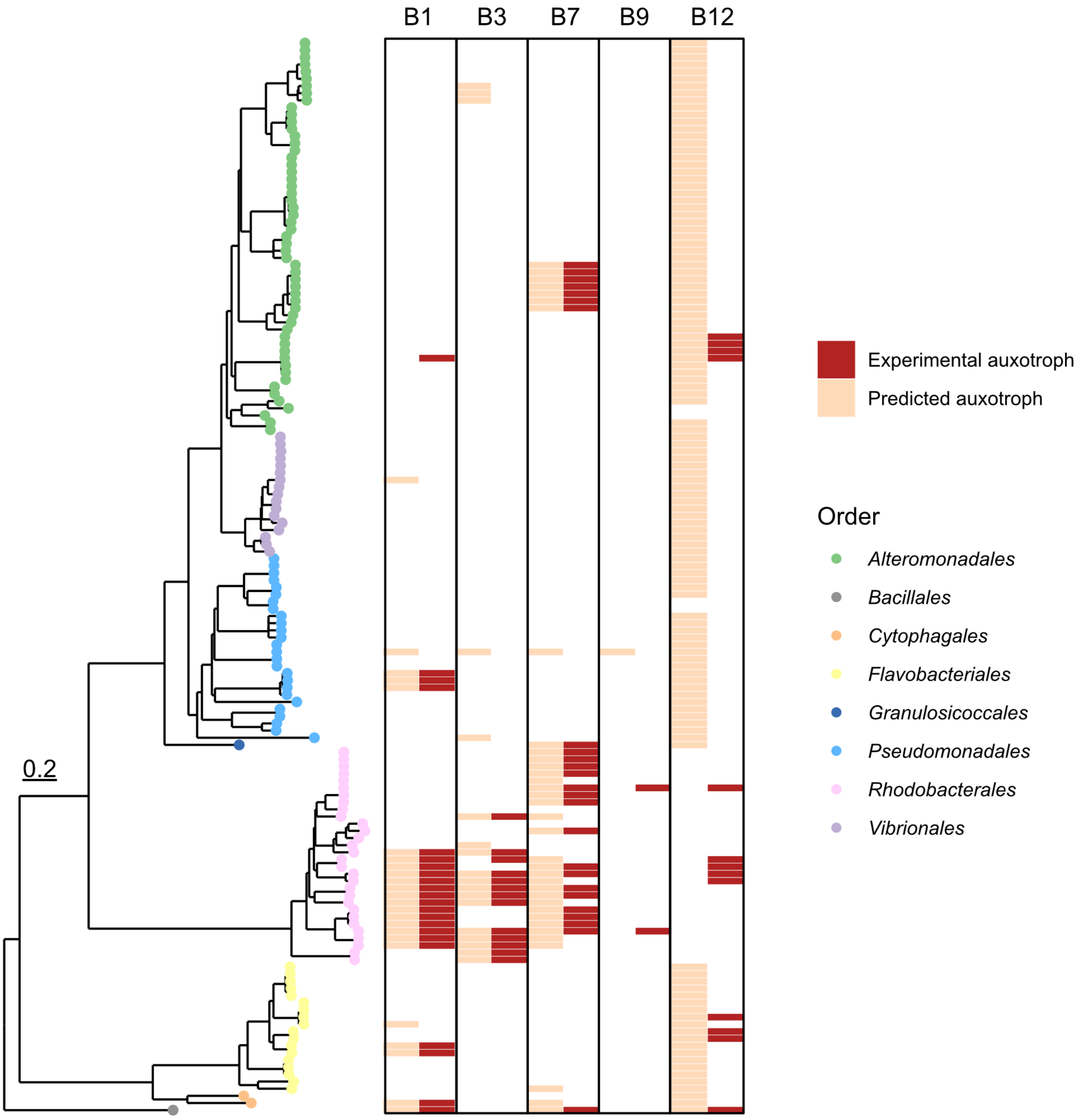

Fig. S4. Phenotype-genotype matching based on presence/absence of key genes (see Table S1).

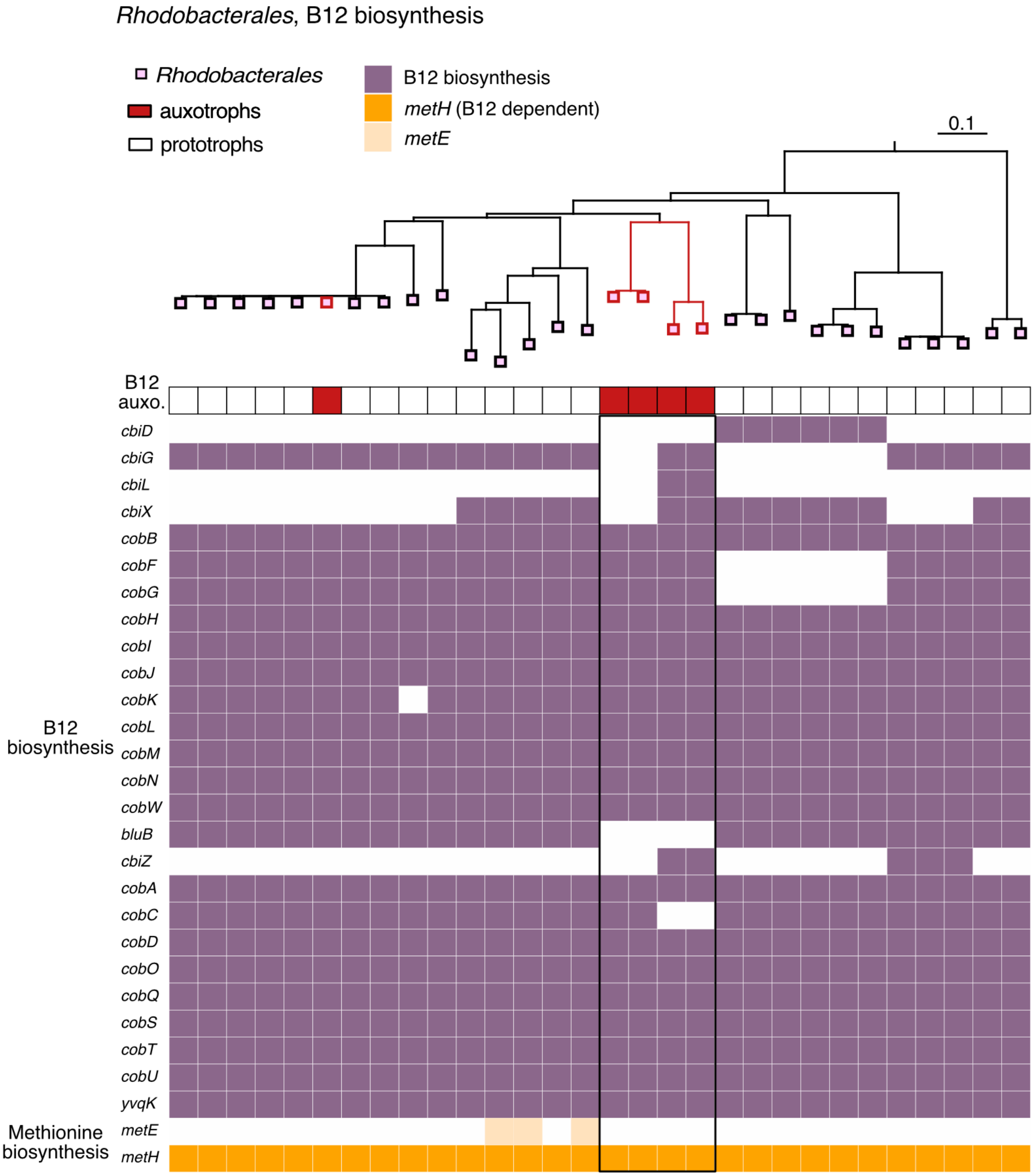

Fig. S5. B12 biosynthesis and dependent genes in *Rhodobacterales*. All but 3 of the Rhodobacterales lack *metE* (peach) and are therefore dependent on B12 for methionine biosynthesis via *metE* (orange). The clade of four auxotrophs outlined in black are likely precursor auxotrophs for 5,6-dimethylbenzimidazole (DMB), as they are missing *bluB* [8]. However, since they retain most of the pathway, they are misclassified as prototrophs based on gene predictors (Table S1).

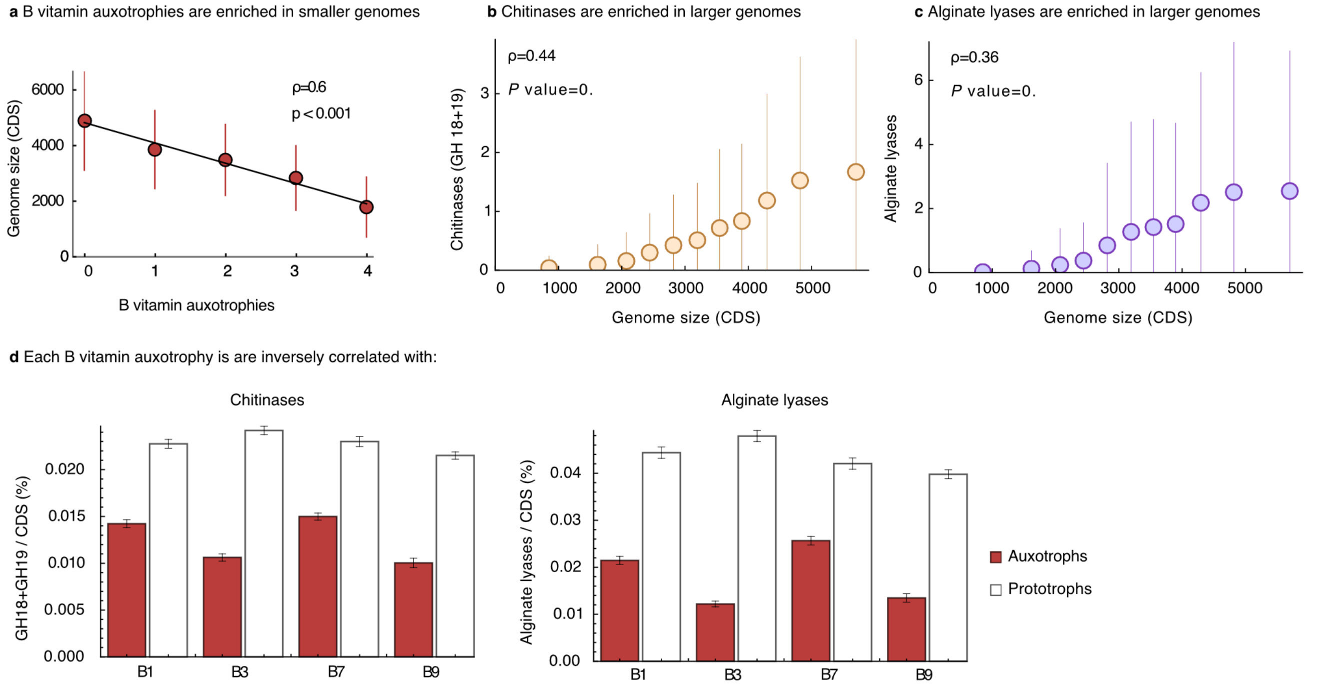

Fig. S6. Vitamin prototrophy is positively correlated with chitin and alginate degradation genes across 11,000 diverse reference genomes from proGenomes. (A) Auxotrophies are enriched in smaller genomes. The number of auxotrophies for vitamins B1, B3, B7, and B9 were assigned based on presence-absence of key biosynthesis genes (Table S1). (B and C) Polysaccharide degradation genes are enriched in larger genomes for both chitin (B) and alginate (C). (D) Even when normalized by genome size (coding sequences, CDS) there is a positive correlation between vitamin prototrophies and chitinases (left) and alginate lyases (right). All *P* values are derived from unconstrained linear model fits.

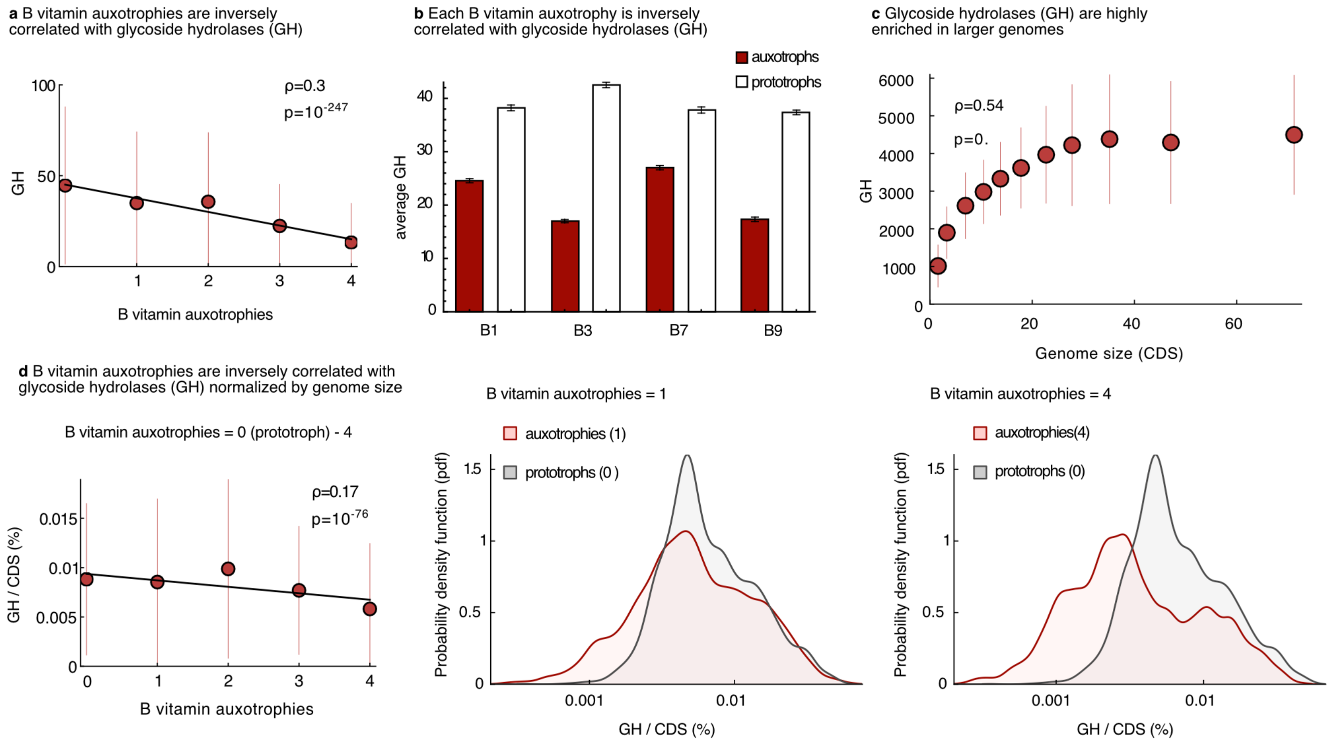

Fig. S7. Vitamin prototrophy is positively correlated with glycoside hydrolase (GH) genes across 11,000 diverse reference genomes from proGenomes. (A) B vitamin auxotrophies are inversely correlated with GH genes. Genomes with more B vitamin auxotrophies have fewer GH genes. (B) Each B vitamin auxotrophy is associated with a lower number of GH genes on average in genomes. (C) GH genes are highly enriched in larger genomes, as was shown for chitinases and alginate lyases (Fig. S6). The opposite is true for vitamin auxotrophies, which are enriched in smaller genomes (Fig. S6.) (D) Left: Genomes with more B vitamin auxotrophies have fewer GH genes, even when normalized by genome size (coding sequences, CDS). GHs as a percent of total genome size are enriched in prototrophs compared to auxotrophs for one B vitamin (center, P value$<10^{-36}$, Mann-Whitney test), especially when compared to auxotrophs for all four vitamins (right, P value $<10^{-151}$, Mann-Whitney test). All P values are derived from unconstrained linear model fits.

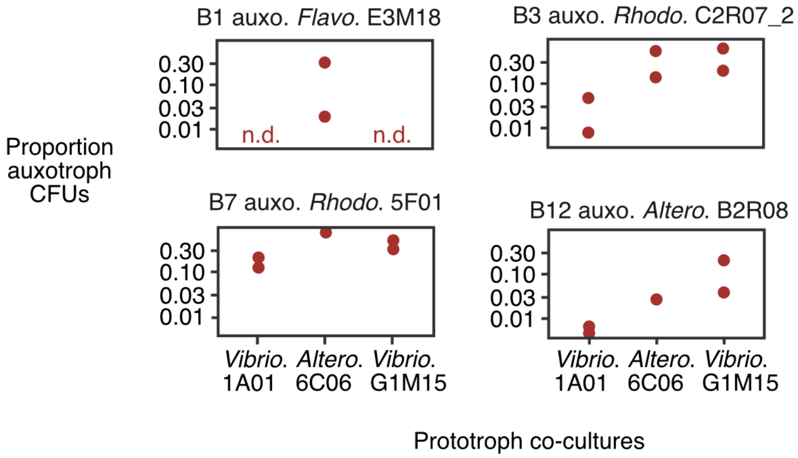

Fig. S8. Auxotroph-prototroph co-cultures. The co-cultures were performed as in Fig. 4A. Auxotrophs and prototrophs were grown separately with vitamins, then combined 1:1 and transferred to fresh medium without vitamins. The media contained four carbon sources (glucose, glutamine, glycerol, and pyruvate) to enable the growth of isolates with different carbon preferences (see Methods). Growth was measured after 3 growth-dilution cycles. Auxotroph growth could not be directly measured by OD600, since all cultures contained prototrophs and grew to high optical densities even in the absence of vitamins. Therefore, we selected four auxotrophs that form pigmented colonies and could be differentiated from the prototrophs, and the ratio between auxotrophs and prototrophs was measured by counting colony forming units (CFUs). Cultures were measured in duplicate (two replicates were removed due to contamination for 5F01-6C06 and B2R08-6C06).

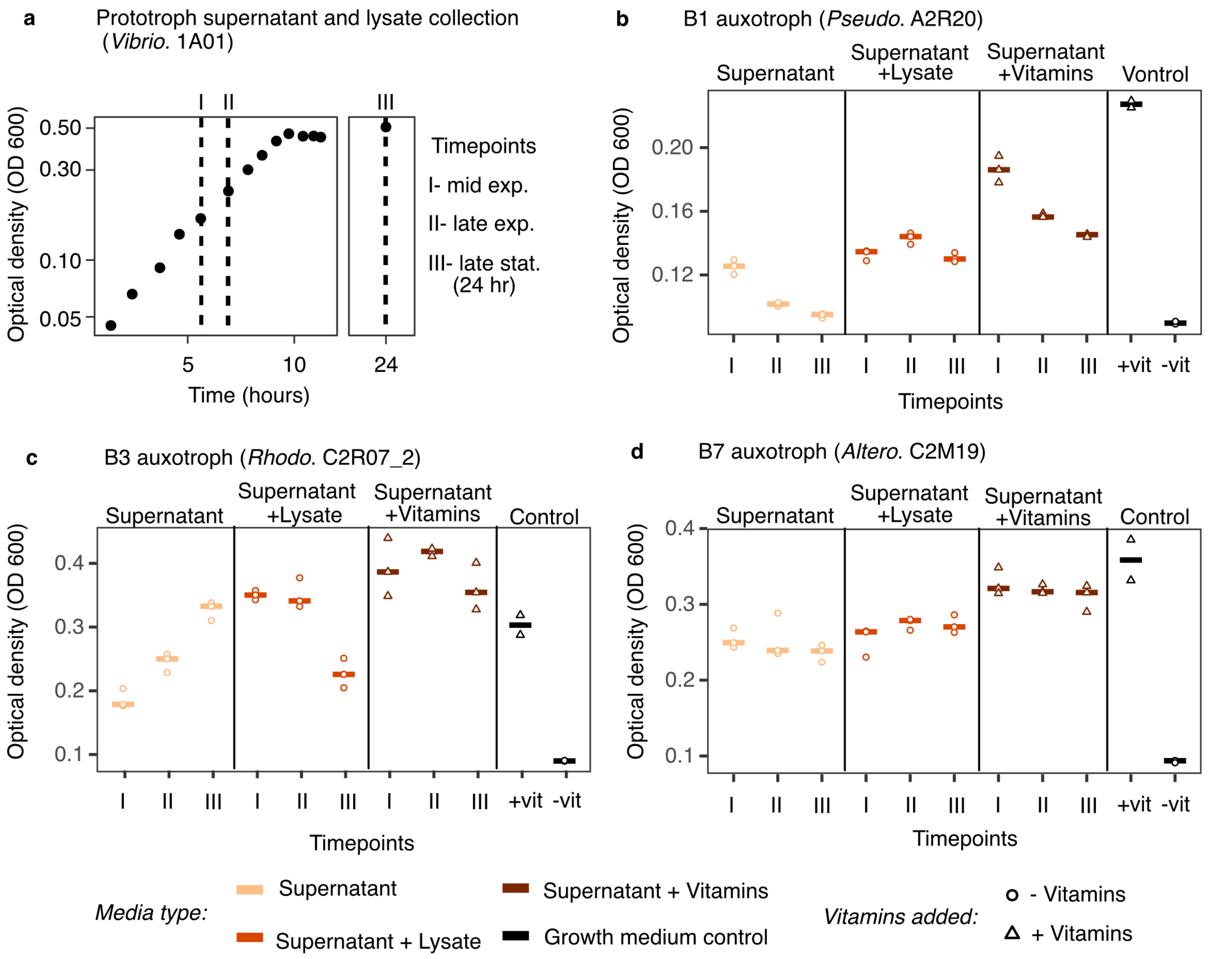

Fig. S9. Auxotroph growth on prototroph supernatants and lysates. (A) Supernatants and lysates were collected from a *Vibrionales* 1A01 prototroph culture in three growth stages: mid and late exponential phase, and late stationary phase (24 hours). At each timepoint, half of the culture was used to generate sterile supernatant (peach), and the other half was harvested to generate sterile supernatant+lysate: cells were lysed in the culture using a probe sonicator, producing a mix of supernatant and lysate, which was then centrifuged and filter sterilized (red). (B-D) Auxotrophs were grown on each supernatant and supernatant+lysate, as well as controls: supernatant supplemented with vitamins (brown) and media controls with and without vitamins (black). The growth of the auxotrophs was dependent on the vitamin auxotrophy as well as the growth stage of the prototroph.

Note: In some cases, the growth on supernatants or lysates with vitamin supplementation was higher or lower than in fresh medium plus vitamins (black, control +vit), indicating that additional nutrients are being produced or depleted. To account for this, all data in Fig. 4B are normalized by the supernatant+vitamin control in each timepoint, to obtain the percentage of growth without vitamin limitation in each condition. Normalized data from timepoint II are presented in Fig. 4B.

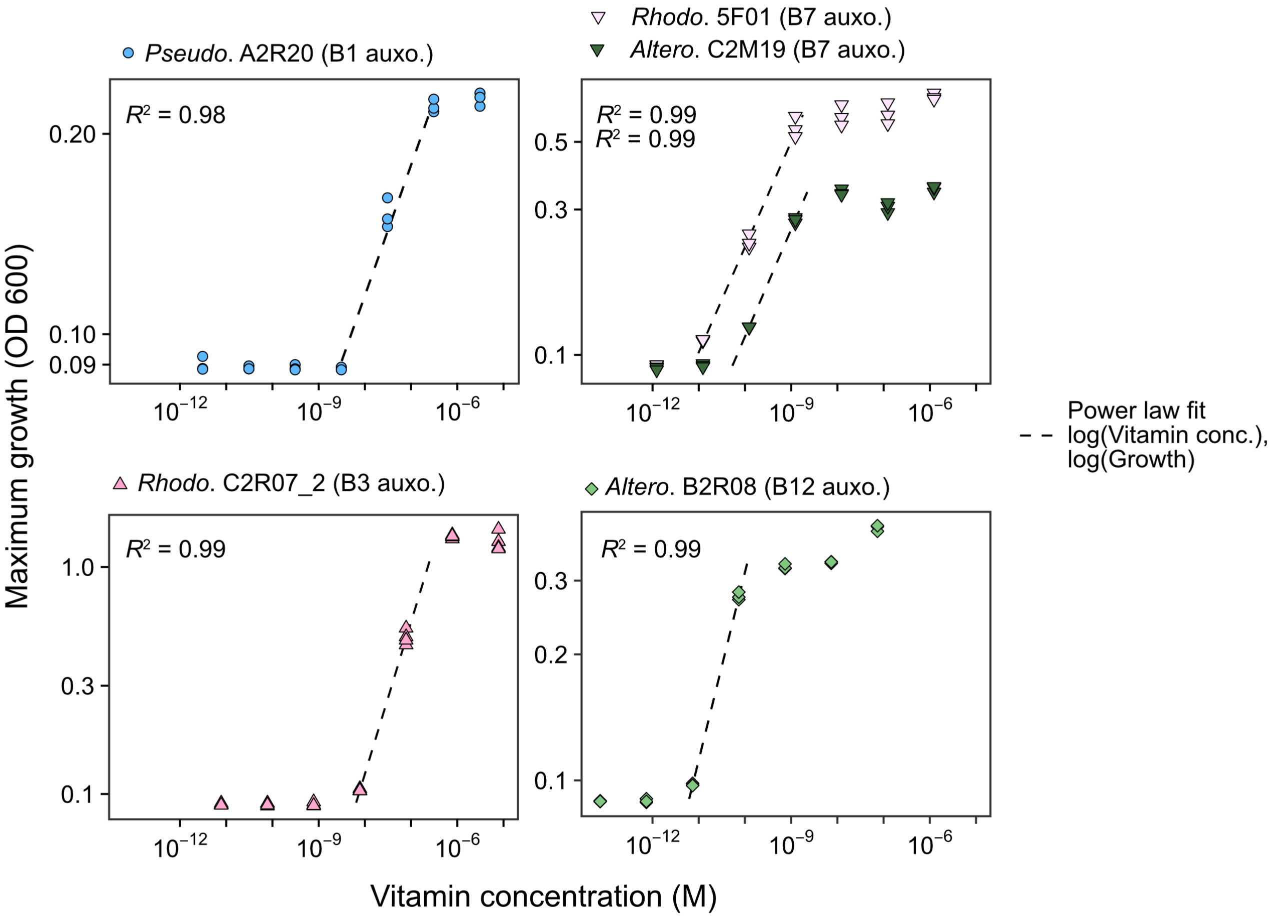

Fig. S10. Maximum growth of auxotrophs as a function of vitamin concentration. Maximum growth for each vitamin concentration was measured as part of the growth rate experiment (Fig. 3). Each auxotroph exhibited three growth regimes as a function of vitamin concentration: no detectable growth; a sharp increase in growth; and saturation (at which point we presume vitamins are no longer limiting). In the regime of growth increase, we found a power law fit between vitamin concentration and growth (dashed lines). For two auxotrophs, A2R20 and 5F01, there were three vitamin concentrations in the regime of sharp increase in growth (in triplicate, nine data points in total); for other auxotrophs, there were two points (in triplicate, six data points in total). A coefficient of determination (R^2) was calculated for all fits.

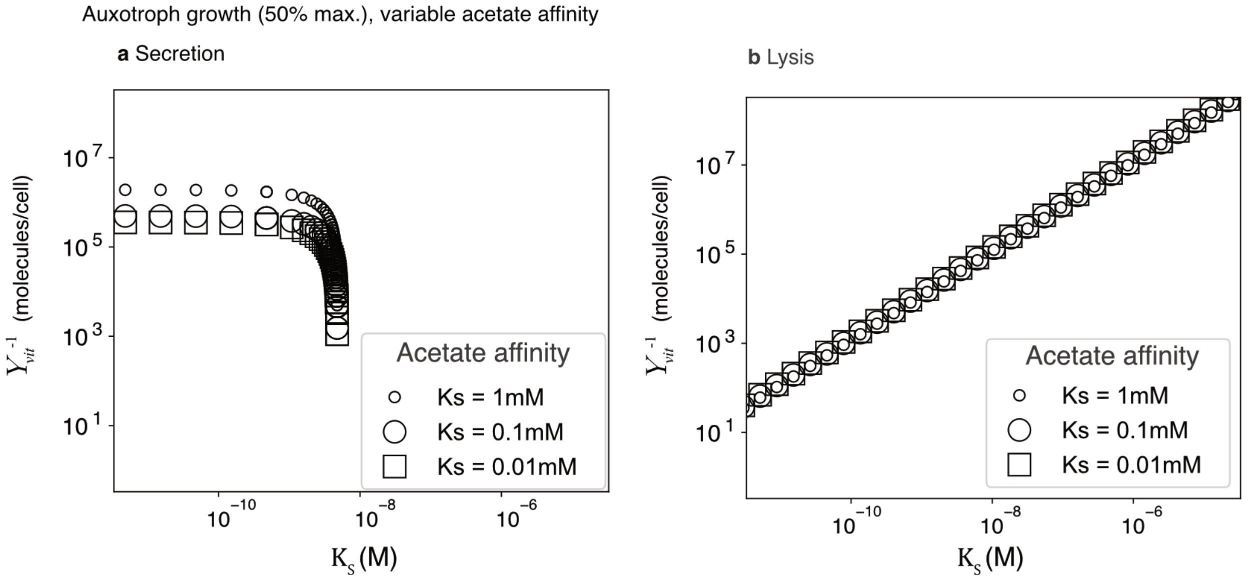

Fig. S11. Model parameters: acetate affinity constant. Here, we use 1 mM, based on measurements in *E. coli* [4–6] (small circles). Marine bacteria are slower growing and may have lower affinities for acetate (large circles, squares). However, this parameter has little effect on growth via secretion, (a) and no perceptible effect on growth via lysis (b).

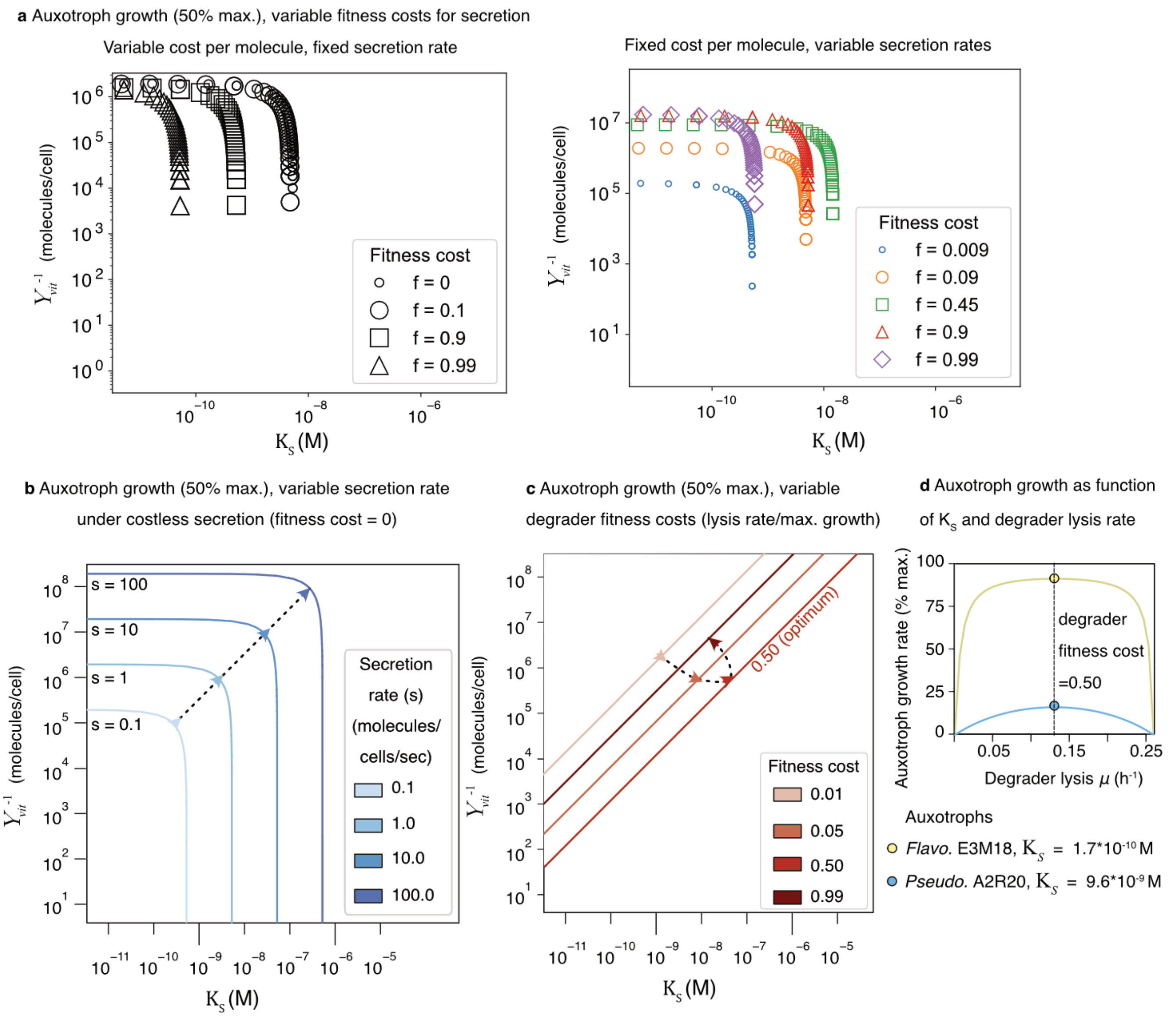

Fig. S12. Model parameters: fitness costs, secretion rates, and lysis rates. (A) Effect of varying fitness costs for secretion. At a fixed secretion rate, even a sizable fitness cost of 0.1 (left) has almost no effect on the growth by secretion, although much higher costs do lead to a lower threshold value for K_S. At a fixed fitness cost per molecule, increasing secretion rates facilitates growth and shifts the 50% auxotroph growth contour line towards the top-right quadrant of the phase space. This continues until the overall fitness cost approaches an optimum value close to 0.5. After this point, increasing secretion rates becomes too costly for degraders, leading to a decrease in secreted vitamin levels and hindering auxotroph growth. (B) Increasing the secretion rate (under costless secretion) leads to increased auxotroph growth. (C) Increasing the lysis rate (i.e., fitness cost) leads to an optimum at fitness cost 0.50, i.e. when degrader lysis rate is equal to half of the maximum degrader growth rate (here, 0.26 hour^{-1}). (D) Auxotroph growth as a function of degrader lysis varies based on the auxotroph's Km (yellow and blue circles) but is always optimal at fitness cost 0.50, i.e. half the maximum degrader growth rate (0.26 h^{-1}), representing a decrease from 9 to 4.5 doublings a day.

Table S1. Auxotrophy predictors based on presence/absence of key biosynthesis genes.

Vit.	Essential biosynthesis genes	Reference
B1	*thiC* and [*thiG* or *thi4*] and [*thiE* or *thiN*]	Paerl et al 2018 [9]
B3	*nadC* + *nadD* + *nadE* +<br>1. (*nadX* or *nadB*) + *nadA* or<br>2. *kmo*	Magnúsdóttir et al 2015 [10] and Lima et al 2009 [11]<br>Note: *kmo* added as representative gene from kynurenine pathway for quinolinate biosynthesis via tryptophan, present in *Flavobacteriales* [11].
B7	*bioF* + *bioA* + *bioD* + *bioB*	Wienhausen et al 2022 [12]
B9	*folK* + *folP*	de Crécy-Lagard et al 2007 [13]<br>Note: While this predictor did not correctly predict auxotrophies for the experimental auxotrophs, there were only two experimental auxotrophs for B9, making it difficult to assess whether this is generalizable. Alternate predictors relying on all genes in the pathway, e.g. Magnúsdóttir et al 2015 [10], resulted in the majority of prototrophs misclassified as auxotrophs, and were therefore not used.
B12	Aerobic biosynthesis (23 total): ALA synthesis (either EC:2.3.1.37 or both EC:1.2.1.70 and EC:5.4.3.8), EC:4.2.1.24, EC:2.5.1.61, EC:4.2.1.75, EC:2.1.1.107, EC:2.1.1.130, EC: 1.14.13.83, EC:2.1.1.131, EC:2.1.1.133, EC:2.1.1.152, EC:1.3.1.54, EC:2.1.1.132, EC:5.4.99.61, EC:6.3.5.9, EC:6.6.1.2, EC:2.5.1.17, EC:6.3.5.10, EC:6.3.1.10, EC:2.7.1.156, cobinamide activation (EC:2.7.7.62), cobalamin phosphatase (EC:3.1.3.73), EC:2.7.8.26<br>Tetrapyrrole precursor biosynthesis (5 total): ALA synthesis (either EC:2.3.1.37 or both EC:1.2.1.70 and EC:5.4.3.8), EC:4.2.1.24, EC:2.5.1.61, EC:4.2.1.75, EC:2.1.1.107	Shelton et al 2019 [14]<br>Note: B12 producers were classified based on this reference as very likely cobamide producers containing all 23 genes in the aerobic pathway, as well as likely cobamide producers containing ≥4/5 tetrapyrrole precursor biosynthesis steps and ≥90% aerobic pathway steps. While genes from the anaerobic pathway of B12 biosynthesis were checked as well, this pathway did not add any additional predicted producers in this dataset.

Table S2. Summary of auxotrophy predictions from genomes compared to phenotypes.

					Mismatches (%) *Per phenotype*	Mismatches (%) *Overall*
B1	Auxo.	Auxo.	Match	21	Auxotrophs: 4.5	Overall B1: 2.7
	Auxo.	Proto.	Mismatch	1		
	Proto.	Proto.	Match	125	Prototrophs: 2.3	
	Proto.	Auxo.	Mismatch	3		
B3	Auxo.	Auxo.	Match	12	Auxotrophs: 7.7	Overall B3: 4.7
	Auxo.	Proto.	Mismatch	1		
	Proto.	Proto.	Match	131	Prototrophs: 4.4	
	Proto.	Auxo.	Mismatch	6		
B7	Auxo.	Auxo.	Match	25	Auxotrophs: 0	Overall B7: 6.7
	Auxo.	Proto.	Mismatch	0		
	Proto.	Proto.	Match	115	Prototrophs: 8	
	Proto.	Auxo.	Mismatch	10		
B9	Auxo.	Auxo.	Match	0	Auxotrophs: 100	Overall B9: 2
	Auxo.	Proto.	Mismatch	2		
	Proto.	Proto.	Match	147	Prototrophs: 0.7	
	Proto.	Auxo.	Mismatch	1		
B12	Auxo.	Auxo.	Match	8	Auxotrophs: 38.5	Overall B12: 75.3
	Auxo.	Proto.	Mismatch	5		
	Proto.	Proto.	Match	29	Prototrophs: 78.8	
	Proto.	Auxo.	Mismatch	108		

Table S3. Power law fits of maximum auxotroph growth (OD 600) as a function of vitamer concentration ([Vit]), including coefficients of determination (R^2) and number of datapoints. See Fig. S10 for plots.

Strain	Auxo.	Power law fit	R^2	Datapoints
A2R20	B1	$\log_{10}$([Vit]) = [5.00654067]*$\log_{10}$(OD 600) + -3.3084900989856703	0.9849	9
C2R07_2	B3	$\log_{10}$([Vit]) = [1.48216213]*$\log_{10}$(OD 600) + -6.632175478123212	0.9967	6
5F01	B7	$\log_{10}$([Vit]) = [2.85080899]*$\log_{10}$(OD 600) + -8.16839469031912	0.9938	9
C2M19	B7	$\log_{10}$([Vit]) = [2.8456014]*$\log_{10}$(OD 600) + -7.323574877066893	0.9991	6
B2R08	B12	$\log_{10}$([Vit]) = [2.22159703]*$\log_{10}$(OD 600) + -8.88757850678276	0.9994	6

Table S4. Concentration of vitamers in 1A01 supernatants and lysates.

Strain	Auxo.	1A01 Sample	Median growth (OD 600)	Estimated vitamer concentration (M)[a]	Ratio of vitamers in lysate:supernatant	Estimated vitamer secretion rate (s) (molecules/cell/sec)[b]
A2R20	B1	Supernatant+lysate	0.1441	6.24E-08	4.89	2.58
		Supernatant	0.1017	1.06E-08		
		Lysate	N/A	5.18E-08		
C2R07_2	B3	Supernatant+lysate	0.3411	9.62E-08	0.59	14.7
		Supernatant	0.2501	6.05E-08		
		Lysate	N/A	3.57E-08		
C2M19	B7	Supernatant+lysate	0.2787	2.48E-09	0.54	0.39
		Supernatant	0.2392	1.61E-09		
		Lysate	N/A	8.70E-10		

[a] Vitamer concentrations were calculated based on auxotroph growth as a function of vitamer concentration (see equations in Table S3). Auxotrophs were grown in two conditions: supernatants alone, and a mixture of supernatants and lysates (Fig. 4). The vitamer concentration in the lysates was estimated by subtracting the concentration in the supernatant conditions from the supernatant and lysate conditions.

[b]See SI text, section II for calculation of secretion rates.

Table S5. Model parameters.

Model parameter name	Value	Reference or formula
Cross-feeder maximum growth rate (r_{cf})	$r_{cf} = 0.3h^{-1}1$	Average rates on range of substrates for marine isolate collection in this study [2]
Yield on acetate (Y_{act})	$31\ OD/M_{act}$	Value measured for marine isolate 3B05 growing on acetate [3]
CFU/ml to OD conversion	$9 \cdot 10^8 \frac{CFU/ml}{OD}$	Value measured for marine isolate 1A01 growing on glucose [3]
Yield on vitamins (molecules per cell)	20,000 (B1); 100,000 (B3); 100 (B7); 5,000 (B12)	Approximated based on published measurements [12, 15, 16]
Affinity on acetate ($K_{s/m}$)	1 mM	General order of magnitude taken from *E. coli* [4–6]
Affinity on vitamins ($K_{s/v}$)	Variable	Measured in this study (Table 2)
Diffusion constant of substrate (D_M)	$12 * 10^{-6} cm^2/s$	The diffusion constant for acetate was used in all simulations
Diffusion constant of vitamins (D_V)	$3.67 * 10^{-6} cm^2/s$	The diffusion constant for B12 was used in all simulations
Degrader death rate (μ)	$\mu = 0 - 0.26h^{-1}$	Free parameter
Degrader secretion rate (s)	$s = 0 - 100\ molecules/s$	Free parameter
Fitness cost due to secretion (f)	$f = c \cdot s = 0 - 1$	Free parameter
Degrader maximum growth rate (r)	$r = 0.26h^{-1}$	Value measured for marine isolate 1A01 growing on chitin [1]
Equilibrium density of degraders with $\mu = 0$ (σ_0)	$7\ cells/\mu m^2$	$\sigma_0 = \rho_b \eta \frac{V}{N_c 4\pi R_0^2}$ as previously in [1]
Density of a monolayer of cells (ρ)	$0.5\ cells/\mu m^3$	Estimated from approximate cell volume of $2\ \mu m^3$
Equilibrium density of degraders (σ_{eq})	$\sigma_{eq} = 0 - 7\ cells/\mu m^2$	$\sigma_{eq} = \left(1 - \frac{\mu}{r}\right)\sigma_0$
Specific activity of chitinases (k_e)	$24\ nmol_{Gln}/\mu g_e/h$	[1]
Enzyme per biomass on particle surface (${Y_e}^{-1}$)	$25\ \mu g_e/OD/ml$	[1]
Acetate produced per GlcNAc consumed ($f_{act/gln}$)	$1.48\ mol_{Act}/mol_{Gln}$	Flux balance analysis (FBA) for 1A01 [7]
Flux of acetate from particle surface per cell density (F_{act}/σ_{eq})	$1.65 \cdot 10^5 \frac{molecules/s}{CFU}$	$\frac{k_e f_{act/gln}}{Y_e}$
Flux of vitamins from particle surface (F_{vit})	Variable	$F_{vit} = \frac{\sigma_{eq}}{Y_{vit}}\mu + \sigma_{eq}s$

Table S6**.** Numerical and analytical estimates for auxotroph growth rates on particles.

Strain (auxo.)	Analytical approximation of I	Proportion of max. analytical growth (%) (r_{auxo}/r_{max})	Numerical estimation of I	Proportion of max. numerical growth (%) (r_{auxo}/r_{max})
A2R20 (B1)	0.180	15.3	0.179	15.2
E3M18 (B1)	10.1	91.0	9.94	90.9
B2R09 (B3)	0.00187	0.2	0.00187	0.2
C2R07_2 (B3)	0.233	18.9	0.232	18.8
5F01 (B7)	0.0663	6.2	0.0662	6.2
C2M19 (B7)	0.0278	2.7	0.0278	2.7
B2R08 (B12)	15.4	93.9	15.1	93.8

Dataset S1 (separate file). List of isolates (Excel file).

Dataset S2 (separate file). Environmental vitamin measurements from literature (Excel file).

Supporting References

1. Guessous G, Patsalo V, Balakrishnan R, et al. Inherited chitinases enable sustained growth and rapid dispersal of bacteria from chitin particles. *Nat Microbiol* 2023; **8**: 1695–1705.
2. Gralka M, Pollak S, Cordero OX. Genome content predicts the carbon catabolic preferences of heterotrophic bacteria. *Nat Microbiol* 2023; **8**: 1799–1808.
3. Amarnath K, Narla AV, Pontrelli S, et al. Stress-induced metabolic exchanges between complementary bacterial types underly a dynamic mechanism of inter-species stress resistance. *Nature Communications* 2023; **14**: 3165.
4. Kotte O, Volkmer B, Radzikowski JL, et al. Phenotypic bistability in Escherichia coli's central carbon metabolism. *Mol Syst Biol* 2014; **10**: 736.
5. Wolfe AJ. The acetate switch. *Microbiol Mol Biol Rev* 2005; **69**: 12–50.
6. Bhattacharya SK, Uberoi V, Dronamraju MM. Interaction between acetate fed sulfate reducers and methanogens. *Water Res* 1996; **30**: 2239–2246.
7. Iffland-Stettner A, Okano H, Gralka M, et al. A genome-scale metabolic model of marine heterotroph Vibrio splendidus strain 1A01. *mSystems* 2023; **8**: e0037722.
8. Taga ME, Larsen NA, Howard-Jones AR, et al. BluB cannibalizes flavin to form the lower ligand of vitamin B12. *Nature* 2007; **446**: 449–453.
9. Paerl RW, Sundh J, Tan D, et al. Prevalent reliance of bacterioplankton on exogenous vitamin B1 and precursor availability. *Proc Natl Acad Sci U S A* 2018; **115**: E10447–E10456.
10. Magnúsdóttir S, Ravcheev D, de Crécy-Lagard V, et al. Systematic genome assessment of B-vitamin biosynthesis suggests co-operation among gut microbes. *Front Genet* 2015; **6**: 148.
11. Lima WC, Varani AM, Menck CFM. NAD biosynthesis evolution in bacteria: lateral gene transfer of kynurenine pathway in Xanthomonadales and Flavobacteriales. *Mol Biol Evol* 2009; **26**: 399–406.
12. Wienhausen G, Bruns S, Sultana S, et al. The overlooked role of a biotin precursor for marine bacteria - desthiobiotin as an escape route for biotin auxotrophy. *ISME J* 2022.

13. de Crécy-Lagard V, El Yacoubi B, de la Garza RD, et al. Comparative genomics of bacterial and plant folate synthesis and salvage: predictions and validations. *BMC Genomics* 2007; **8**: 1–15.

14. Shelton AN, Seth EC, Mok KC, et al. Uneven distribution of cobamide biosynthesis and dependence in bacteria predicted by comparative genomics. *ISME J* 2019; **13**: 789–804.

15. Sultana S, Bruns S, Wilkes H, et al. Vitamin B12 is not shared by all marine prototrophic bacteria with their environment. *The ISME Journal* 2023; **17**: 836–845.

16. Bruns S, Wienhausen G, Scholz-Böttcher B, et al. Simultaneous quantification of all B vitamins and selected biosynthetic precursors in seawater and bacteria by means of different mass spectrometric approaches. *Anal Bioanal Chem* 2022; **414**: 7839–7854.
